# Time to negative throat culture following initiation of antibiotics for pharyngeal group A *Streptococcus*: a systematic review and meta-analysis up to October 2021 to inform public health control measures

**DOI:** 10.2807/1560-7917.ES.2023.28.15.2200573

**Published:** 2023-04-13

**Authors:** Emma McGuire, Ang Li, Simon M Collin, Valerie Decraene, Michael Cook, Simon Padfield, Shiranee Sriskandan, Chris Van Beneden, Theresa Lamagni, Colin S Brown

**Affiliations:** 1 United Kingdom Health Security Agency (UKHSA), London, United Kingdom; 2 NIHR Health Protection Research Unit in Healthcare-associated Infection and Antimicrobial Resistance, Imperial College London, Hammersmith Hospital Campus, London, United Kingdom; 3 CDC Foundation, Atlanta, Georgia, United States

**Keywords:** *Streptococcus pyogenes*, Group A Strep Infection, Streptococcal, Infectious Disease Outbreaks, Disease Transmission, Infectious

## Abstract

**Background:**

Public health guidance recommending isolation of individuals with group A streptococcal (GAS) infection or carriage for 12–24 h from antibiotic initiation to prevent onward transmission requires a strong evidence base.

**Aim:**

To estimate the pooled proportion of individuals who remain GAS culture-positive at set intervals after initiation of antibiotics through a systematic literature review (PROSPERO CRD42021290364) and meta-analysis.

**Methods:**

We searched Ovid MEDLINE (1946–), EMBASE (1974–) and Cochrane library. We included interventional or observational studies with ≥ 10 participants reporting rates of GAS throat culture positivity during antibiotic treatment for culture-confirmed GAS pharyngitis, scarlet fever and asymptomatic pharyngeal GAS carriage. We did not apply age, language or geographical restrictions.

**Results:**

Of 5,058 unique records, 43 were included (37 randomised controlled studies, three non-randomised controlled trials and three before-and-after studies). The proportion of individuals remaining culture-positive on day 1, day 2 and days 3–9 were 6.9% (95% CI: 2.7–16.8%), 5.4% (95% CI: 2.1–13.3%) and 2.6% (95% CI: 1.6–4.2%). For penicillins and cephalosporins, day 1 positivity was 6.5% (95% CI: 2.5–16.1%) and 1.6% (95% CI: 0.04–42.9%), respectively. Overall, for 9.1% (95% CI: 7.3–11.3), throat swabs collected after completion of therapy were GAS culture-positive. Only six studies had low risk of bias.

**Conclusions:**

Our review provides evidence that antibiotics for pharyngeal GAS achieve a high rate of culture conversion within 24 h but highlights the need for further research given methodological limitations of published studies and imprecision of pooled estimates. Further evidence is needed for non-beta-lactam antibiotics and asymptomatic individuals.

## Introduction

*Streptococcus pyogenes* (group A *Streptococcus*, GAS) causes a range of clinical syndromes from mild infections such as impetigo, pharyngitis and scarlet fever to life-threatening invasive group A streptococcal (iGAS) infection. Severe infections predominantly affect elderly people, infants and women in the first month after giving birth [1,2]. GAS may also colonise the skin or pharynx of asymptomatic people. The prevalence of asymptomatic GAS throat carriage is approximately 7% (95% CI: 5.6–8.8) globally, with higher rates reported in children and young adults under 20 years of age (8%, 95% CI: 6.6–9.7) [3]. However, reported rates in adults rarely exceed 1% in the United Kingdom (UK) [4,5]. Colonisation may be transient or prolonged, and the mechanisms leading to chronic GAS carriage are not fully understood [6].

Outbreaks arise from both symptomatic and asymptomatic index cases and can occur in a range of settings including households, hospitals and care homes [7-9]. Without treatment, the secondary attack rate for pharyngitis and scarlet fever is estimated to be 25–35% in outbreaks within a family setting and 23–60% in educational setting outbreaks [10-12]. Even with treatment of the index case, attack rates among contacts are high, e.g. 26% among school contacts and 13% among household contacts [7]. In classroom outbreaks, prevalence of the outbreak strain has been noted to increase rapidly among asymptomatic contacts, reaching 27% in week 2 [7]. Within households, the median time between primary and secondary cases is 6 days (range: 0–30 days) [13]. GAS infections are treated with antibiotics to shorten the duration of symptoms and reduce the risk of severe disease or long-term sequelae, including rheumatic fever [14,15]. In GAS outbreaks, antibiotic treatment is recommended for individuals both with symptomatic infection and with asymptomatic carriage to induce bacteriological clearance and prevent onward transmission. The importance of treatment in reducing transmission was highlighted by the recent UK experience. Relatively high rates of scarlet fever and iGAS were observed in 2022 and research demonstrated that household cases of scarlet fever cases have an increased risk of invasive disease, and that strains causing superficial and invasive infection are genetically identical [16-18].

Current public health guidance recommends that patients with GAS pharyngitis or scarlet fever stay home from work, school or daycare until at least 12–24 h (United States) and 24 h (UK) after starting antibiotic treatment, and some US guidelines also recommend exclusion until resolution of symptoms [19-22]. Antibiotic chemoprophylaxis is also recommended for high-risk asymptomatic close contacts of iGAS cases [23]. Despite the impact of isolation from school or work, evidence for how long infectivity is likely to persist once treatment has commenced has not been reviewed systematically. We aimed to fill this evidence gap by reviewing studies which reported time from initiation of antibiotics to negative GAS culture in patients with confirmed pharyngeal GAS infection or carriage.

## Methods

### Search strategy and selection criteria

Our protocol was registered with PROSPERO (CRD42021290364) on 8 November 2021. A professional librarian (MC) searched Ovid MEDLINE (1946–), EMBASE (1974–) and the Cochrane library on 18 October 2021 without language restrictions (see Supplementary Appendix A for search terms). Bibliographies of systematic reviews identified were searched for eligible studies. Studies included met the following criteria (see Supplementary Appendix B for full criteria): (i) peer-reviewed primary research with 10 or more participants; (ii) patients with GAS pharyngitis, scarlet fever or asymptomatic pharyngeal GAS carriage confirmed by positive throat culture according to methods in ‘*Laboratory diagnosis of group A streptococcal infections*’ [24] or which reported diagnosis specifically of ‘group A’ streptococcal infection; (iii) antibiotics administered to participants; (iv) reported rates or counts of patients with positive or negative throat cultures for GAS during antibiotic treatment, or time to clearance following initiation of antibiotics. Animal or in vitro studies, case reports, letters, commentaries and conference abstracts were excluded as were duplicate reports of the same raw data. 

Two reviewers (EM, AL) independently screened titles and abstracts using the rayyan.ai platform [25]. Risk of bias was assessed independently and in parallel by two reviewers (EM, AL) using US National Institutes of Health study quality assessment tools [26]. At all stages, disagreements were resolved by discussion between the two reviewers with arbitration, if required, by a third reviewer (CB, TL).

### Data analysis

Data from each study were extracted by one reviewer (EM) and cross-checked by a second reviewer (AL) into a custom Microsoft Excel form. The main data items extracted were: characteristics of study (design, location), patients (age, sex), treatment (drug, administration route, dose and duration), GAS identification method, and frequencies of study participants who were tested and who were culture-positive on day 1, day 2 and days 3–9 from start of antibiotic treatment. For the primary outcome of time to negative throat culture on antibiotic treatment, we conducted random effects meta-analysis to calculate pooled proportions of study participants who remained culture-positive at each time point. Where studies reported culture results at other time points, we mapped these to the later corresponding time point. For example, a study reporting on days 1–2 would be included in the day 2 pooled estimate.

All statistical analyses were performed using STATA version 15.0 (StataCorp). The score method was used to produce study specific 95% confidence intervals (CIs) and the Freeman-Tukey double arcsine transformation was used to calculate weighted pooled proportion estimates for each sub-group [27]. The Wald method was used to produce CIs for these pooled estimates and heterogeneity within subgroups was tested using the chi-squared test and quantified by the *I* ^2^ statistic. Meta-analysis was performed for all antibiotics combined and for subgroups by type of antibiotic (classes and individual types) using metaprop_one [28].

For the secondary outcome of culture positivity after completing antibiotic therapy, the same approach was used to calculate pooled proportions of participants who were culture-positive in the early (< 72 h), intermediate (72 h–10 days) and late (> 10 days) post-antibiotic period. Where studies reported typing, meta-analysis was performed using the same approach to calculate pooled proportions or participants who had documented clearance of GAS at the end of therapy followed by either relapse or reacquisition of the same strain of GAS or acquisition of a new strain of GAS after antibiotics, by class of antibiotic. The same approach was used to calculate pooled proportions reporting adverse drug reactions, by class of antibiotic. Meta-regression of proportion against day of culture (as ordinal variable) was performed using metapreg (to test for linear trend) and metareg (to generate bubble and line plots) [29,30].

## Results

**Figure 1:**
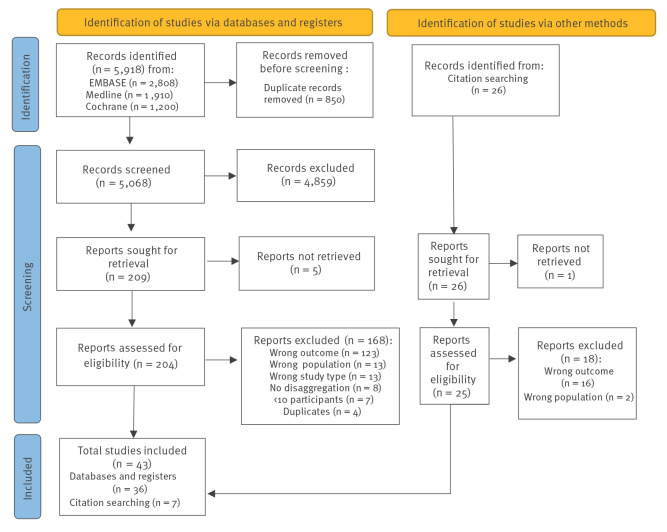
PRISMA flow diagram for strategy of identification, screening and inclusion of studies reporting group A *Streptococcus* culture positivity following initiation of antibiotics for pharyngeal group A *Streptococcus,* up to October 2021

Database searches identified 5,068 unique records (after exclusion of 850 duplicates) of which 209 were selected for full text review. Five full texts could not be obtained, and 168 studies were excluded (Figure 1). Citation searches identified a further 26 studies, of which 25 were retrieved and 18 were excluded. 

### Study characteristics

Overall, 43 studies published between 1958 and 2015 were included in the review, representing observations on 7,168 patients with GAS confirmed on throat culture. Most were randomised controlled trials (n = 37), three were non-randomised controlled trials, and three were observational ‘before and after’ studies (Table). A minority reported early culture results taken 24 h (n = 12) or 48 h (n = 9) after antibiotic initiation. Most reported outcomes only in children (n = 29), with 11 studies in children and adults and two in adults only; age group was not reported in one study. Pharyngitis (n = 42) and tonsillitis (n = 12) were the most studied clinical diagnoses, with few reporting on scarlet fever (n = 5) or asymptomatic carriage (n = 3). Most reported on the use of penicillins (n = 34) or cephalosporins (n = 14). Studies were predominantly conducted in North America (n = 33) and Europe (n = 6). Six studies were assessed as having a ‘low’ risk of bias, 20 as having a ‘moderate’ risk of bias and 17 as having a ‘high’ risk of bias (see Supplementary Appendix C for risk of bias assessments of included studies).

**Table:** Characteristics of included studies reporting group A *Streptococcus* culture positivity following initiation of antibiotics for pharyngeal group A *Streptococcus* up to October 2021 (n = 42)

Author [ref.]	Country	Study design	GAS n	Age mean years (range)	Male n (%)	Female n (%)	Clinical	Treatment	Routine culture time during antibiotic course	Routine culture time after antibiotic course	In vitro GAS identification method
Schalet et al. 1958 [60]	US	NRS	90	18 (17–27)	90 (100)	0 (0)	P/T	Buffered penicillin G 250,000 IU TDS for 10 days, Penicillin V 200,000 IU TDS for 10 days	Day 1, day 2, day 3^a^, day 4^a^ and day 5^a^	Day 10 ^a^, day 12^a^, day 15^a^, day 17^a^ and day 21^a^	Lancefield grouping, Unspecified protein typing
Edmond et al. 1966 [59]	US	NRS	77	12.4 (7–18)	41 (53)	36 (47)	P (67%) /A (33%)	Penicillin V 125 mg TDS for 7 days, Penicillin G 125 mg TDS for 7 days	18–24 h, day 2 and day 3	Day 10^a^, day 14^a^, day 21^a^, day 28^a^, day 35^a^ and 2–3 months^a^ post-therapy	Bacitracin sensitivity, Lancefield grouping
Aronovitz et al. 1968 [85]	US	RCT	100	NR (2–16)	NR	NR	P/T	Dicloxacillin 6 mg/lb/day QDS for 10 days, Penicillin V 12 mg/lb/day QDS for 10 days	Day 4	Day 10 and day 21	Unspecified method
Howie et al. 1971 [54]	US	RCT	228	NR (NR)	NR	NR	P/A	Lincomycin < 22.7 kg 750 mg/day TDS, 22.7–44.9 kg 1g/day TDS, > 45.0 kg 1,250 mg/day TDS for 10 days, Penicillin G 750,000 IU/day TDS for 10 days, ^b^Benzathine penicillin G 600,000 IU if < 59 lb, 900,000 IU if 60–89 lb, 1,200,000 IU if ≥ 90 lb IM single dose	Day 5	Day 14, day 31 and day 60^a^	Bacitracin sensitivity, Fluorescent Lancefield grouping, T-protein typing, M-protein typing
Azimi et al. 1972 [86]	US	BAS	49	NR (1–15)	17 (68)	32 (65)	P	Cephalexin infant < 1 year old 125 mg QDS, ≥ 1 year old 250 mg QDS for 10 days	Days 1–2	Day 10, day 17, day 24 and day 31	Bacitracin sensitivity
Levine et al. 1972 [36]	US	RCT	99	NR (1–16)	NR	NR	P/S (7%)	Clindamycin 16 mg/kg/day TDS or QDS for 10 days, Erythromycin 16 mg/kg/day TDS/QDS for 10 days	Days 2–6	Day 10 and days 20–31	Unspecified method
Colcher et al. 1972 [55]	US	RCT	300	NR (1–15)	NR	NR	P	Penicillin V 250 mg TDS for 10 days, Penicillin V 250 mg TDS for 10 days (+ parental counselling), ^b^Benzathine penicillin/procaine penicillin G 1.2 million IU IM single dose	Day 9	3–6 weeks post-therapy	Unspecified method
Sinanian et al. 1972 [42]	US		155	NR (NR)	NR	NR	P	Penicillin V 125 QDS if < 50lb or 250 QDS if weight ≥ 50 lb for 10 days, Clindamycin 75 mg QDS to 150 mg TDS if < 55 lb, 150 mg TDS to 150 mg QDS if 55 lb–75 lb, or 150 mg QDS to 300 mg QDS if > 75lb for 10 days, ^b^Clindamycin 75 mg QDS to 150 mg TDS if < 55 lb, 150 mg TDS to 150 mg QDS if 55 lb–75 lb, or 150 mg QDS to 300 mg QDS if > 75 lb for 5 days	Day 7	Day 14 and day 30	Unspecified method
Ryan et al. 1973 [39]	Australia	RCT	110	NR (1–15)	NR	NR	P	Erythromycin estolate 30–50 mg/kg/day QDS for 10 days, Erythromycin stearate 30–50 mg/kg/day QDS for 10 days	Day 1, day 2, day 3	Days 13–21	Bacitracin sensitivity, Lancefield grouping, T-protein typing
Trickett et al. 1973 [43]	US	RCT	87	NR (NR)	NR	NR	P/T	Co-trimoxazole 2 tablets BD (80/400 mg) for 10 days, Buffered penicillin G 250 mg QDS for 10 days	Day 2 and day 4	Day 10^a^, day 14^a^, day 21^a^ and day 28^a^	Fluorescent Lancefield grouping
Rabinovitch et al. 1973 [87]	Canada	RCT	118	NR (NR)	NR	NR	P	Penicillin V or G 250,000–400,000 IU QDS for 10 days, Cephalexin 125 mg QDS for 10 days	Day 7	Day 10	Bacitracin sensitivity
Lester et al. 1974 [34]	US	RCT	628	NR (NR)	NR	NR	P	Penicillin V 500 mg/day if < 22.6 kg, 1 g/day if > 22.7 kg QDS for 14 days, ^b^Benzathine penicillin 600,000 IU if < 27.3 kg, 900,000 IU if 27.4–40.9 kg, 1,200,000 IU if > 41 kg IM single dose, Erythromycin 500 mg/day if < 22.6 kg, 1 g/day if > 22.7 kg QDS for 14 days Clindamycin palmitate 150 mg/day if 8.2–17.2 kg, 300 mg/day if 17.3–34 kg, 600 mg/day if 34.1–68.2 kg QDS for 14 days, Clindamycin HCL 300 mg/day QDS if < 24.9 kg, 450 mg/day TDS if 25–34 kg, 600 mg/day QDS if > 34.1 kg for 14 days, Clindamycin HCL 300 mg/day if < 24.9 kg, 600 mg/day BD if > 25 kg for 14 days	Day 5	Day 14^a^ and day 31^a^	Fluorescent Lancefield grouping, T-protein typing, M-protein typing
Mogabgab et al. 1976 [88]	US	NRS	23	24 (13–52)	8 (35)	15 (65)	P	Cephradine 250 mg QDS for 10 days, Cephalexin 250 mg QDS for 10 days	Days 2–6	None	Lancefield grouping
Ginsburg et al. 1980 [89]	US	RCT	96	6.6 (2–14)	44 (46)	52 (54)	P/T	Penicillin V 8 mg/kg QDS for 10 days, Cefadroxil 15 mg/kg BD for 10 days	Day 5	Day 14 and day 31	Bacitracin sensitivity, Lancefield grouping, T-protein typing, M-protein typing
Schwartz et al. 1981 [57]	US	RCT	210	NR (1–18)	NR	NR	P/S	Penicillin V 20 mg/kg/day if < 50 kg and 15 kg/kg/day if ≥ 50 kg all TDS for 7 days, Penicillin V 20 mg/kg/day if < 50 kg and 15 kg/kg/day if ≥ 50 kg all TDS for 10 days	Days 4–6	Day 12–14^a^, days 20–22^a^ and days 27–30^a^	Bacitracin sensitivity, unspecified Lancefield method, M-protein typing
Hoskins et al. 1981 [44]	UK	RCT	30	NR (11–17)	30 (100)	0 (0)	A	Co-trimoxazole 2 tablets BD (125 mg/375 mg) for 7–10 days	Days 3–4	Days 10–12^a^	Unspecified method
Ginsburg et al. 1982A [33]	US	RCT	175	8 (2–16)	91 (52)	84 (48)	P	Erythromycin estolate 15 mg/kg/day BD for 10 days, Erythromycin succinate 15 mg/kg/day BD for 10 days	Day 5	Day 14 and days 17–31	Bacitracin sensitivity, T-protein typing, M-protein typing
Ginsburg et al. 1982B [40]	US	RCT	198	7.5 (2–15)	100 (51)	98 (49)	P/T	Penicillin V 8 mg/kg TDS for 10 days, Cefadroxil 15 mg/kg BD for 10 days, Erythromycin 15 mg/kg BD for 10 days, ^b^Benzathine penicillin/procaine penicillin G 900,000 IU/300,000 IU IM single dose	Day 5	Day 14 and days 17–31^a^	Bacitracin sensitivity, Lancefield grouping, T-protein typing, M-protein typing
Randolph et al. 1985 [51]	US	RCT	194	8.8 (2–20)	NR	NR	P	Penicillin V 250 mg TDS for 10 days, Cefadroxil 250 mg TDS for 10 days, ^b^Placebo	18–24 h	None	Bacitracin sensitivity
Krober et al. 1985 [53]	US	RCT	21	9.3 (6–17)	4 (19)	17 (81)	P	Penicillin V 250 mg TDS for 10 days, ^b^Placebo	Day 1, day 2 and day 3	None	Bacitracin sensitivity
Stillerman et al. 1986 [90]	US	RCT	104	NR (NR)	53 (51)	51 (49)	P	Cefaclor 20 mg/kg/day for 10 days, Penicillin V 20 mg/kg/day for 10 days	Days 2–4 and days 8–10^d^	Days 12–14^a^, weekly for 2 weeks^a^	Bacitracin sensitivity, Lancefield grouping, T-protein typing, M-protein typing
Gerber et al. 1986 [91]	US	RCT	555	8 (1–25)	NR	NR	P	Cefadroxil 15 mg/kg/day BD or 30 mg/kg OD for 10 days, Penicillin V 250 mg TDS, 8 mg/kg TDS or 8 mg/kg QDS for 10 days, ^b^Benzathine penicillin 9,000,000 IU + procaine penicillin 300,000 IU IM single dose, ^b^Erythromycin 15 mg/kg BD for 10 days	18–24 h	Day 15^a^, day 24^a^ and day 41^a^	Bacitracin sensitivity, Lancefield grouping
Gerber et al. 1987 [92]	US	BAS	128	NR (3–21)	NR	NR	P	Penicillin V (dose not specified) for 10 days	18–24 h	Days 14–16	Bacitracin sensitivity, Lancefield grouping
Pavesio et al. 1988^c^ [56]	Italy	RCT	60	5 (2–11)	32 (53)	28 (47)	P/T/S	Ceftriaxone 50 mg/kg OD IV for 1–3 days	Day 1	Days 16–19^a^	Unspecified method
Gerber et al. 1989 [45]	US	RCT	154	9.8 (3–21)	82 (53)	72 (47)	P	Penicillin V 250 mg TDS for 10 days, Penicillin V 750 mg OD for 10 days	18–24 h	Days 14–16 and days 24–31	Bacitracin sensitivity, Lancefield grouping
Milatovic et al. 1989 [93]	Germany	RCT	269	NR (NR)	NR	NR	P/T	Penicillin V 25,000 IU/kg TDS - max 1.2 million IU/day for 10 days, Cefadroxil 25 mg/kg BD for 10 days	Days 3–5	Days 11–15 and days 21–35	Lancefield grouping
Krober et al. 1990 [46]	US	RCT	142	7.9 (3–18)	71 (50)	71 (50)	P	Penicillin V 1,000 mg OD for 10 days, Penicillin V 500 mg BD for 10 days, Penicillin V 250 mg QDS for 10 days	Day 2	Day 4 and days 5–18	Unspecified serological method
Disney et al. 1990 [38]	US	RCT	208	12.2 (1–68)	95 (46)	113 (54)	P/T	Cefadroxil 30 mg/kg/day OD for 10 days, Erythromycin 30 mg/kg/day QDS for 10 days	Days 7–8	Days 13–16 and days 28–30	Unspecified method
Stein et al. 1991 [35]	US	RCT	89	28.5 (12–58)	NR	NR	P	Clarithromycin 250 mg BD for 10 days, Penicillin V 250 mg QDS for 10 days	Days 4–6	Days 13–15 and days 28–34	Unspecified serological method
Block et al. 1992 [94]	US	RCT	101	9.1 (3–18)	NR	NR	P	Cefixime 8 mg/kg OD if < 50 kg, 400 mg OD if ≥ 50 kg for 10 days, Penicillin V 250 mg TDS for 10 days	Days 3–7	Days 12–17^a^, 3 weeks^a^ and 6 weeks^a^ post-therapy	Bacitracin sensitivity, Lancefield grouping, T-protein typing, M-protein typing
De la Garza et al. 1992 [41]	US	RCT	45	13 (13)	NR	NR	P/T	Cefprozil dose not standardised but max dose was 500 mg/day if < 25 kg for 10 days, Erythromycin 30 mg/kg/day 10 days	Day 1 and days 7–10^d^	Days 14–16 and days 28–30	Unspecified method
Shvartzman et al. 1993 [49]	Israel	RCT	157	NR (>3)	64 (41)	93 (59)	P	Penicillin V 250 mg TDS or QDS for 10 days, Amoxicillin children 50 mg/kg/day, adults 750 mg OD for 10 days	Day 2	Day 14^a^	Bacitracin sensitivity
Snellman et al. 1993 [31]	US	RCT	47	8.9 (4–16)	33 (70)	14 (30)	P	Erythromycin 250 mg TDS for 10 days, Penicillin V 250 mg TDS for 10 days, ^b^Benzathine penicillin 600,000 or 1.2 million IU (by weight) IM single dose	18–24 h	None	Bacitracin sensitivity, T-protein typing, M-protein typing
Pichichero et al. 1994 [95]	US	RCT	377	7.8 (2–17)	NR	NR	P/T	Cefpodoxime 10 mg/kg/day BD for 10 days, Cefpodoxime 10 mg/kg/day OD for 5 days, Penicillin V 40 mg/kg/day TDS for 10 days	Days 3–5	Days 9–12^a^, days 14–17 and days 32–38	Unspecified serological method
Raz et al. 1995 [47]	Israel	RCT	104	26.7 (12–66)	39 (38)	65 (63)	P	Penicillin V 1 g BD for 10 days, Penicillin V 500 mg QDS for 10 days	Day 9	Day 38	Bacitracin sensitivity, Lancefield grouping
Pacifico et al. 1996 [58]	Italy	RCT	179	6.8 (3–12)	75 (42)	104 (58)	P/S (30%)	Penicillin V 50,000 units/kg BD for 10 days, ^b^Azithromycin 10 mg/kg OD for 3 days	Days 4–5	Days 12–14 and days 34–36	T-protein typing
Dagnelie et al. 1996 [52]	The Netherlands	RCT	239	25.6 (4–59)	93 (39)	146 (61)	P	Penicillin V 250 mg for 4 to 9-year-olds, 500 mg for those 10 years or older TDS for 10 days, ^b^Placebo	Day 2	None	Lancefield grouping
Watkins et al. 1997 [37]	US	RCT	257	28.6 (over 12)	NR	NR	P	Dirithromycin 500 mg OD for 10 days, Penicillin V 250 mg QDS for 10 days	Days 3–5	Days 13–15, and 3–5 weeks post-therapy	Unspecified serological method
Feder et al. 1999 [48]	US	RCT	152	9.9 (5–12)	97 (64)	55 (36)	P	Penicillin V 250 mg TDS for 10 days, Amoxicillin 750 mg OD for 10 days	Day 1	Days 14–16 and days 24–31	Lancefield grouping
Esposito et al. 2002 [67]	Italy	RCT	348	5.5 (2–14)	179 (51)	169 (49)	P	Amoxicillin 40 mg/kg/day TDS for 10 days, ^b^Cefaclor 40 mg/kg/day BD for 5 days	Days 6–7	Day 11–15, day 16–20 and day 28–35	T-protein typing
Lennon et al. 2008 [50]	New Zealand	RCT	353	NR (5–12)	178 (50)	175 (50)	P/T	Amoxicillin 1,500 mg OD (or 750 mg if < 30 kg weight) for 10 days, Penicillin V 500 mg BD (or 250 mg if < 20 kg weight) for 10 days	Days 3–6	Days 12–16 and days 26–36	Bacitracin sensitivity, M-protein typing
Brook et al. 2009 [65]	US	BAS	50	NR (4–16)	27 (54)	23 (46)	P/T	Amoxicillin 40 mg/kg or 250 mg TDS for 10 days, Cefdinir 14 mg/kg or 600 mg OD for 10 days	Day 1, day 2, day 3 and day 4	Day 10 and day 12	Bacitracin sensitivity, Lancefield grouping
Schwartz et al. 2015 [32]	US	RCT	111	6.7 (2–17)	66 (59)	45 (41)	P/S (3%)	Amoxicillin 50 mg/kg single dose	Day 1	None	Lancefield grouping

A: asymptomatic carriage; BAS: before and after study; BD: twice daily; IU: international units; NR: not reported; NRS: non-randomised study; OD: once daily; P: pharyngitis; QDS: four times daily; RCT: randomised controlled trial; ref.: reference number; S: scarlet fever; T: tonsillitis; TDS: three times daily.

^a^ Time points not used in meta-analysis as results reported were not disaggregated.

^b^ Study arm not used in meta-analysis as did not meet inclusion criteria (e.g. cultures taken only after antibiotic course, culture results not reported for these arms, or not disaggregated).

^c^ Study not included in meta-analysis, as this was the only study which used intravenous antibiotics.

^d^ Analysed within the day 3–9 group.

Study methodologies were broadly similar; routine culture for beta-haemolytic streptococci was performed, most commonly on blood agar, with preliminary and confirmatory GAS identification. Throat swabs were repeated at pre-defined visits after initiation of treatment. Only reporting of bacterial culture for GAS was used to determine the primary and secondary outcomes in this review.

### Culture positivity during antibiotic treatment

**Figure 2:**
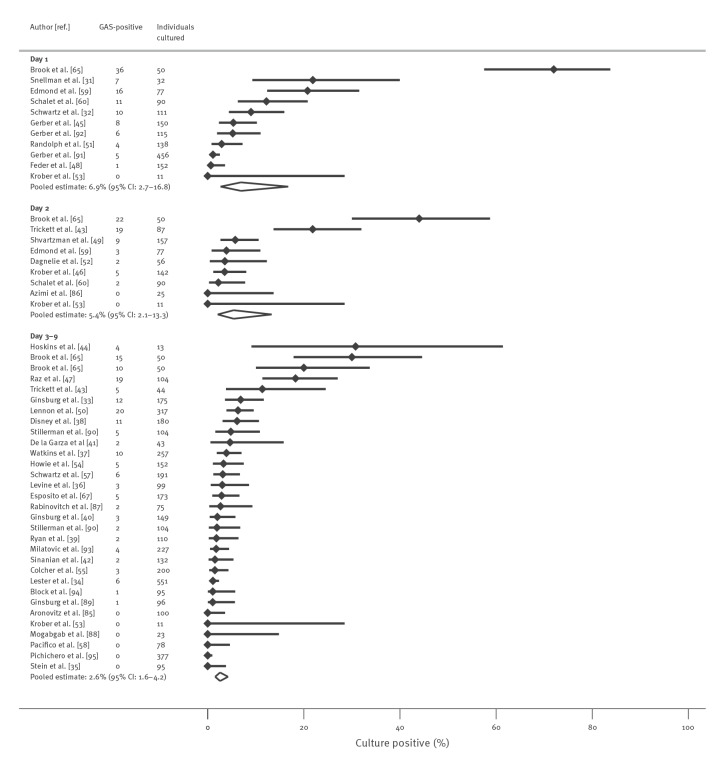
Proportion of patients with culture-confirmed group A streptococcal throat infection or carriage at day 1, day 2 and days 3–9 after treatment initiation with all antibiotic types, up to October 2021 (n = 42 studies)

CI: confidence interval; GAS: group A *Streptococcus*; ref.: reference number.

For all oral antibiotics combined (n = 42 studies), the pooled proportion of individuals with confirmed GAS who remained culture-positive was 6.9% (95% CI: 2.7–16.8) within 24 h after starting treatment (day 1), 5.4% (95% CI: 2.1–13.3) on day 2, and 2.6% (95% CI: 1.6–4.2) between days 3–9 (Figure 2). Brook et al. was an outlier, reporting high proportions of culture-positive patients at 24 h (72.0%) and 48 h (44.0%). Heterogeneity statistics and evidence of differences between sub-group pooled estimates are summarised in Supplementary Appendix E. Brook et al. was a relatively small study and excluding its results changed the summary estimates only slightly, as shown in Supplementary Appendix F.

For each included study, the proportion of individuals who were culture positive (dots) and 95% confidence intervals (CI, lines) are given. The Freeman-Tukey double arcsine transformation was used to calculate weighted pooled proportion estimates for each sub-group (diamond). The Wald method was used to produce CIs for these pooled estimates and heterogeneity within subgroups was tested using the chi-squared test and quantified by the *I* ^2^ statistic.

**Figure 3:**
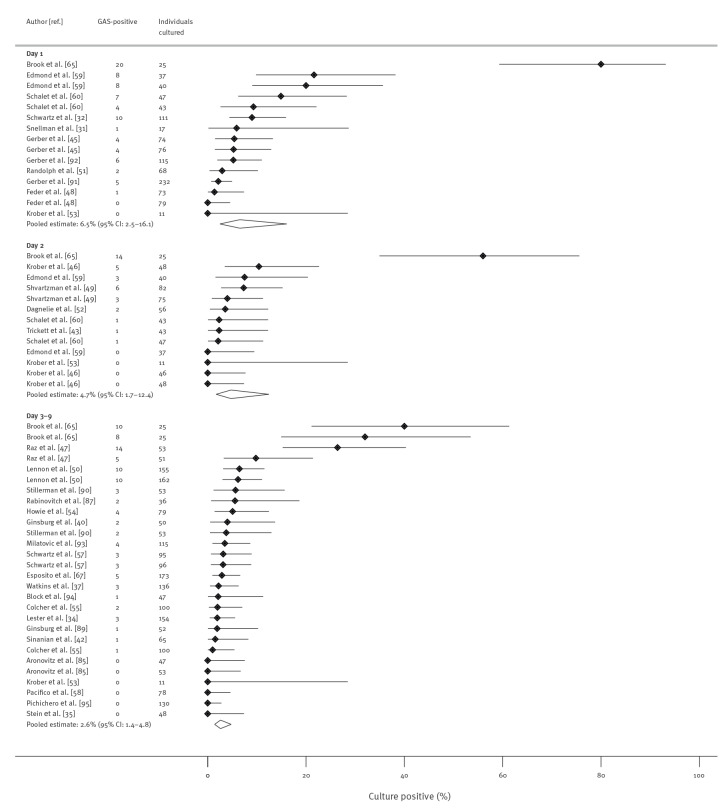
Proportion of patients with culture-confirmed group A streptococcal (GAS) throat infection or carriage at day 1, day 2 and days 3–9 after treatment initiation with penicillin, up to October 2021 (n = 34 studies)

CI: confidence interval; GAS: group A *Streptococcus*; ref.: reference number.

**Figure 4:**
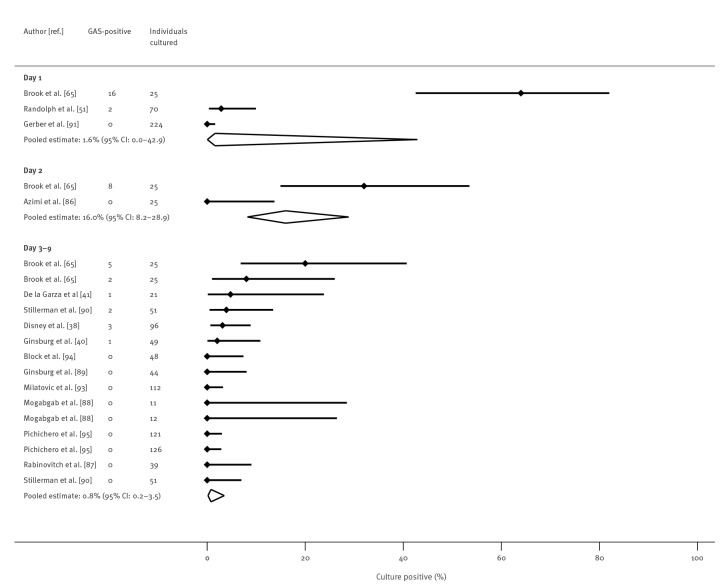
Proportion of patients with culture-confirmed group A streptococcal (GAS) throat infection or carriage at day 1, day 2 and days 3–9 after treatment initiation with cephalosporin, up to October 2021 (n = 14 studies)

CI: confidence interval; GAS: group A *Streptococcus*; ref.: reference number.

Antibiotic class subgroup analyses were possible for penicillins and cephalosporins. For penicillins (Figure 3), positivity was 6.5% (95% CI: 2.5–16.1) on day 1, 4.7% (95% CI: 1.7–12.4) on day 2, and 2.6% (95% CI: 1.4–4.8) on days 3–9. For cephalosporins (Figure 4), positivity was 1.6% on day 1 (95% CI: 0.04–42.9), 16.0% (95% CI: 8.2–28.9) on day 2, and 0.8% (95% CI: 0.2–3.5) on days 3–9. Brook et al. was an outlier reporting high percentage positivity as previously noted. Excluding its results for penicillin changed the summary estimates only slightly (Supplementary Appendix F shows results of sensitivity analyses excluding Brook et al.).

For each included study, the proportion of individuals who were culture positive (dots) and 95% confidence intervals (CI, lines) are given. The Freeman-Tukey double arcsine transformation was used to calculate weighted pooled proportion estimates for each sub-group (diamond). The Wald method was used to produce CIs for these pooled estimates and heterogeneity within subgroups was tested using the chi-squared test and quantified by the *I* ^2^ statistic.

For each included study, the proportion of individuals who were culture positive (dots) and 95% confidence intervals (CI, lines) are given. The Freeman-Tukey double arcsine transformation was used to calculate weighted pooled proportion estimates for each sub-group (diamond). The Wald method was used to produce CIs for these pooled estimates and heterogeneity within subgroups was tested using the chi-squared test and quantified by the *I* ^2^ statistic.

Meta-analysis by single antibiotic agent was possible only for penicillin V. Supplementary Figure S1 showed a day 1 positivity of 4.7% (95% CI: 2.5–8.5), day 2 positivity of 3.8% (95% CI: 2.3–6.3), and day 3–9 positivity of 2.1% (95% CI: 1.2–4.0).

Meta-regression by day of culture did not demonstrate evidence for a linear effect of increasing time on proportion culture-positive for all antibiotics (p = 0.12) nor trends for penicillins (p = 0.21), cephalosporins (p = 0.23) or penicillin V (p = 0.71). Supplementary Figures S2–5 show the meta-regression of the proportion of patients with culture positive confirmed GAS throat infection or carriage on days 1 to 9 after the start of any or specific antibiotic therapy.

Only two studies reported culture results within 24 h of starting therapy. Snellman et al. reported on throat cultures taken on three occasions within the first 24 h [31]. Of the patients who cleared GAS within 24 h, the median time to clearance with any antibiotic (oral penicillin, intramuscular penicillin and oral erythromycin) was 18 h. However, 8/47 (17.0%) patients remained GAS culture-positive at 24 h and their results were not disaggregated by antibiotic. Schwartz et al. reported throat culture results 11–24 h after a single dose of amoxicillin; 10/111 (9.0%) remained culture-positive [32].

Ten studies investigated macrolides [31,33-41], three clindamycin [34,36,42] and two co-trimoxazole [43,44], with a wide range of dosing regimens (Supplementary Appendix D). Only one study reported on throat cultures taken at 24 h following initiation of treatment with a macrolide; 6/15 (40%) remained culture-positive [31]. However, another study reported that 2/110 (1.8%) were culture-positive on 1–3 days after treatment initiation [39]. Regarding lincosamides, we did not identify any studies reporting on cultures taken within 48 h of treatment initiation. However, results from the three studies reporting on later time periods were in line with our overall findings. Two studies reporting on the use of sulphonamides found comparatively high rates of culture positivity; 18/44 (40.9%) and 4/13 (30.8%) remained culture positive on day 2 and days 3–4 following initiation of co-trimoxazole, respectively [43,44].

A wide range of oral penicillin V regimens was used; frequencies ranged from one to four times daily, with a variety of weight or age-based doses in children and adult doses ranging from 500 mg to 2 g per day. Only three studies directly compared oral penicillin V dosing regimens [45-47]. Gerber et al. compared 750 mg once daily to 250 mg three times daily penicillin V, reporting that after 18–24 h 4/76 (5.3%) patients vs 4/74 (5.4%) patients were culture-positive, respectively. Krober et al. compared three dosing regimens of penicillin V; 1,000 mg once daily vs 500 mg twice daily vs 250 mg four times daily. After 2 days of therapy, 5/48 (10.4%), 0/48 (0%) and 0/46 (0%) had positive throat cultures, respectively. Raz et al. compared penicillin V 1 g twice daily to 500 mg four times daily and reported that cultures taken after 9 days of treatment were positive in 5/51 (9.8%) patients and 14/53 (26.4%) patients, respectively. Similarly, eight cephalosporins were investigated, from first to third generation. There were insufficient results for each regimen to support subgroup meta-analyses by penicillin dosing strategy or cephalosporin type.

Several randomised controlled trials compared the efficacy of once daily amoxicillin against penicillin V with broadly similar rates of culture positivity at equivalent time points [48-50]. One compared penicillin V 250 mg three times daily to amoxicillin 750 mg once daily, with 1/73 (1.4%) and 0/79 (0.0%) culture-positive after 18–24 h, respectively [48]. Another compared penicillin V taken 3 or 4 times daily to daily amoxicillin, reporting culture-positivity on day 1 of 26/82 (7.3%) for penicillin and 3/75 (4%) for amoxicillin [49]. A third study compared 500 mg penicillin V twice daily and once daily amoxicillin, with 10/162 (6.2%) and 10/155 (6.5%) patients found to be culture positive after 3–6 days of antibiotics, respectively [50].

Three studies used placebo control arms [51-53]. Dagnelie et al. reported 2/56 (3.6%) patients taking oral penicillin V remained culture-positive after 48 h, compared with 41/55 (74.5%) of those taking placebo [52]. Similarly, Randolph et al. found that 100% of patients with GAS pharyngitis who received placebo remained positive at 18–24 h, compared with ‘ca 3%’ of those in the oral penicillin and cefadroxil groups [51].

Five studies reported intramuscular penicillin therapy; results were consistent with our findings for antibiotics overall [31,34,40,54,55]. Snellman et al. found 1/15 (6.7%) children were culture-positive at 24 h following single dose benzathine penicillin [31]. Lester et al. reported no positive cases at day 5 among 126 patients treated either with benzathine penicillin or combined benzathine penicillin/procaine penicillin G [34]. Colcher et al. found 3/100 (3%) to be culture-positive at day 9 following a single dose of benzathine penicillin/procaine penicillin G [55].

Only one study was identified that reported the effect of an intravenous (IV) antibiotic [56]. This study investigated the treatment of GAS tonsillitis or scarlet fever in 60 children who received either a single dose or 3 days of IV ceftriaxone 50 mg/kg dose. All patients in both arms were culture-negative 24 h after the first dose of IV ceftriaxone.

### Culture positivity after antibiotic treatment

**Figure 5:**
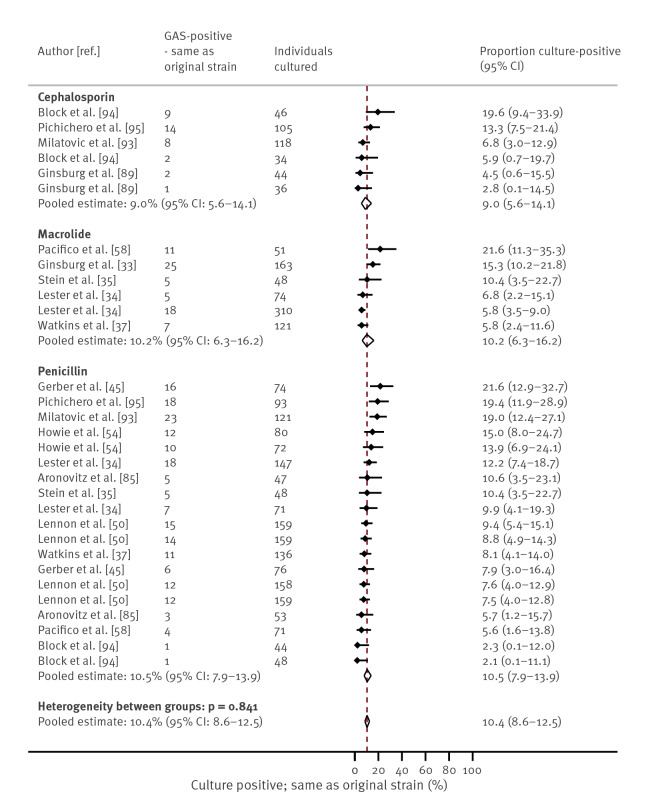
Proportion of patients with culture-confirmed group A streptococcal (GAS) throat carriage^a^ after completion of antibiotic therapy by antibiotic class, where typing was reported, up to October 2021 (n = 13 studies)

CI: confidence interval; GAS: group A *Streptococcus*; ref.: reference number.

In studies in which throat cultures were taken at routine times after completion of antibiotics (n = 23), the proportion of patients who remained culture-positive was 9.1% (95% CI: 7.3–11.3) overall, with 3.1% (95% CI: 1.0–9.5) within 72 h, 8.9% (95% CI: 6.8–11.6) at 72 h to 10 days after completion and 9.9% (95% CI: 7.8–12.7) at 10 or more days (Supplementary Figure S6). Thirteen studies compared the GAS strain identified after completion of antibiotics and initial culture clearance to the original strain, eight by M typing, one by T typing, one by multilocus enzyme electrophoresis and three by an unspecified serotyping method. Among these studies, the overall pooled proportion that had documented culture clearance after therapy followed by a further positive GAS throat culture was 13.3% (95% CI: 10.5–16.7); 10.4% (95% CI: 8.6–12.5) had relapse or reacquisition of the original GAS strain and 2.7% (95% CI: 1.7–4.3) had acquired a new strain of GAS after therapy. For 13 studies where typing was reported to compare strains before and after antibiotics, Figure 5 shows the proportion of patients who were culture positive after antibiotic therapy; Supplementary Figure S7 shows the proportion of patients with relapse or reacquisition of the original GAS strain after antibiotic therapy and S8 shows the proportion of patients with acquisition of a new strain of GAS after antibiotic therapy.

**^a^** including relapse or reacquisition of the same strain and acquisition of a new strain.

For each included study, the proportion of individuals who were culture positive (dots) and 95% CI (lines) are given. The Freeman-Tukey double arcsine transformation was used to calculate weighted pooled proportion estimates for each sub-group (diamond). The Wald method was used to produce CIs for these pooled estimates and heterogeneity within subgroups was tested using the chi-squared test and quantified by the *I* ^2^ statistic.

### Analysis by clinical or patient sub-group

There were insufficient observations to support sub-group meta-analysis by clinical indication (tonsillitis, pharyngitis, scarlet fever or asymptomatic carriage). Five studies included individuals with scarlet fever, but none provided disaggregated results for this sub-group [32,36,56-58]. Three studies included individuals with asymptomatic carriage [44,54,59], but only Hoskins et al. reported distinct results for this sub-group. This was a small paediatric study in which throat cultures were repeated 3–4 days after starting co-trimoxazole, at which point 4/13 (30.8%) children remained culture-positive. As only two studies reported exclusively on adults, meta-analysis by age group was not possible [43,60]. No studies were identified that specifically reported on patients at risk of severe GAS disease, such as pregnant women, older people (aged ≥ 75 years) or immunocompromised individuals. Excluding the results of studies that included asymptomatic carriage (Edmond et al. 1996, 33% of 25 patients; Hoskins et al. 1981, 100% of 30 patients; Howie et al. 1971, unknown proportion of 228 patients) changed the summary estimates only slightly (Supplementary Appendix G).

### Adverse drug reactions

Where reported, the proportion of patients who reported a side effect or adverse drug reaction was 6.0% (95% CI: 2.4–14.3) (n = 14 studies) and the proportion who ceased the study drug due to an adverse reaction was 0.3% (95% CI: 0.1–1.7) (n = 12 studies) (Supplementary Figures S9 and S10).

## Discussion

The key finding of our systematic review and meta-analysis is that antibiotic treatment achieves clearance of pharyngeal GAS in > 90% of individuals 24 h after initiation of therapy. However, GAS was cultured from the pharynx of almost 10% of patients on routine follow-up 10 or more days after completion of antibiotics. Where typing was available, four of five were confirmed to be a relapse or reacquisition of the original GAS strain.

There is no recognised standard for the proportion of individuals who clear GAS that would be considered acceptable. However, a clearance rate of > 90% would appear high considering that studies which included a placebo arm reported that 0–15% of untreated participants were culture-negative at comparable time points [47-49]. Given the high secondary attack rate for GAS and the need to prevent onward transmission, this study provides evidence to support the public health guidelines that recommend that individuals with GAS pharyngitis or scarlet fever isolate for at least 24 h after starting antibiotic treatment. It also supports the need for clearance throat swabs for individuals potentially acting as a reservoir of infection in iGAS outbreaks. The evidence from our review could be ranked at best ‘moderate’, given that half of the included studies were judged to be at moderate risk of bias and only six had low risk of bias. However, these bias assessments applied to the whole of each study, whereas our meta-analyses were based on data from single arms of these trials. Also, our focus is on the overall pattern between days 1, 2 and 3–9 which shows only a gradual decline from 5–7% positivity over days 1–2 to 3% from day 3 onwards. The latter group includes studies demonstrating a persistent level of positivity that is consistent with a precautionary approach to renewed social mixing by individuals with GAS.

Between-study heterogeneity gave rise to overlapping CIs for sub-group estimates at each time point, and meta-regression did not provide evidence that culture-positivity was higher on day 1 than on day 2 or days 3–9 (assuming a linear effect). Our analysis was also unable to discern differences between antibiotic classes or types or between different dosing regimens for the same antibiotic. The primary focus of this study was clearance of pharyngeal GAS during antibiotic treatment and immediately after treatment completion; therefore, the optimal duration of antibiotics for longer term eradication was outside of our scope. However, several reviews have examined this issue [61-64].

There were several outliers among estimates from included studies, notably Brook et al. who conducted a before-and-after study comparing the proportion with positive culture among 50 children with GAS pharyngitis treated with either amoxicillin or cefdinir [65]. While the 40 mg/kg dose of amoxicillin used in this study is lower than the 50 mg/kg recommended in national guidance in the US, this was the same dose used in two other included studies that found much lower rates of culture positivity at comparable time points [48,66,67]. This suggests that methodology rather than underdosing might explain their outlying findings. The Brook et al. study was assessed as having a moderate risk of bias because outcome assessors were not masked and the proportion of eligible patients who were enrolled was unclear. However, other studies had a similar risk of bias; therefore, the reason for their particularly high percentages is unknown. Excluding the results of Brook et al. made only a slight difference to our summary estimates. We mitigated for patients with symptoms being more likely to return for follow-up as a source of bias by only including estimates where all participants were scheduled for repeat specimens, regardless of symptoms. Three quarters of included studies were conducted in North America, and geographical bias should be considered when interpreting these results in other contexts.

Our findings might be affected by antibiotic carry-over, when a high concentration of antibiotic in the sample inhibits in vitro growth [68]. This effect would lower the culture-positive proportion in samples taken during or shortly after the antibiotic course. Additionally, patient non-adherence with antibiotic therapy cannot be ruled out as a factor influencing our findings. Binary culture results may not be an accurate indication of infectiousness. However, quantitative measures which may provide additional value were seldom reported in the included studies.

The appropriate dosing of penicillin V for GAS pharyngitis is unclear. Debate surrounds the effectiveness of twice daily dosing, and more frequent dosing intervals have been associated with reduced adherence [69,70]. The two identified studies in which penicillin V dosing regimens were directly compared suggested that less frequent dosing delivered similar clearance rates, although Gerber et al. considered once daily penicillin V to be inferior to multiple daily dosing with regards to re-acquisition of infection following treatment [45]. Treatment with once daily amoxicillin also appeared to have the same effectiveness as standard multi-dosed penicillin V.

Most patients included in this review were treated with penicillins or cephalosporins, and there remains uncertainty regarding the performance of non-beta-lactam antibiotics, particularly in the early stages of treatment. Unfortunately, studies reporting on macrolides were limited and the only study to report on day 1 culture conversion included only 15 participants in the macrolide arm, with 6 of 15 patients culture-positive 24 h after starting erythromycin [31]. Two meta-analyses evaluated clinical and bacteriological outcomes with clarithromycin, both reporting favourable bacteriological clearance compared with other antibiotics [71,72]. Mass drug administration of single dose azithromycin has been used in the control of iGAS outbreaks among people experiencing homelessness in the US and Canada [73,74]. However, these reviews and outbreak reports assessed bacterial eradication after the antibiotic course.

GAS has retained exquisite in vitro sensitivity to penicillin. However, while the prevalence of *erm* genes conferring resistance to macrolides and lincosamides in GAS bacteraemia in England has remained steady at 8% and 9% respectively, an increasing prevalence of both erythromycin and clindamycin resistance has been reported in iGAS cases in US, reaching 22.8% and 22.0% respectively in 2017 [1,75]. Studies investigating macrolide treatments have explicitly excluded patients with resistant organisms from culture endpoint analysis. Therefore, the prevalence and presence of macrolide resistance is an important consideration when determining optimal treatments. Clindamycin has demonstrated inhibition of GAS virulence factors in vivo and has been recommended as part of combination therapy in severe forms of GAS infection such as necrotising fasciitis and streptococcal toxic shock syndrome for anti-toxin ribosomal effects [76,77]. However, its use in eradicating GAS from the pharynx must be balanced against tolerability considerations. The two studies in our review that investigated co-trimoxazole demonstrated relatively low rates of early culture conversion suggesting this may not be the most effective antibiotic for prevention of GAS transmission in the context of GAS outbreaks.

Although rates of reported penicillin allergy are ca 10%, the prevalence of true penicillin allergy is much lower and rates of anaphylactic penicillin allergy in the UK and US are < 1% [78,79]. Paucity of evidence regarding optimal alternatives such as macrolides and lincosamides requires urgent attention, particularly as resistance varies by region. Evidence to support antibiotic choice for GAS patients with penicillin allergy in settings where macrolide resistance was suspected or present is limited [80]. There were no studies investigating GAS clearance by alternative non-beta-lactam agents such as oxolidazones, fluoroquinolones, tetracyclines, rifampicin, tetracycline, chloramphenicol, glycopeptides or lipoglycopeptides. Antibiotics were generally well tolerated regardless of class. Although rates of reported side effects were higher in non-beta-lactam antibiotics, cessation of the study drug because of side effects was rare for any class of antibiotic.

Presence of symptoms could hypothetically be correlated with culture clearance because inflammation can influence tissue perfusion and antibiotic concentrations. However, only one study investigated effectiveness of clearing asymptomatic GAS carriage and no firm conclusions could be drawn as this was a small study with a sub-optimal antibiotic [44]. This highlights an important evidence gap relating to the use of antibiotics to decolonise asymptomatic contacts identified as potential reservoirs of GAS transmission in outbreak situations. Similarly, the evidence base for optimal treatment of patients with scarlet fever is limited.

Only studies reporting on pharyngeal GAS were reviewed and our results cannot be generalised to infections at other sites. We excluded studies examining the efficacy of antibiotics in individuals with recurrent GAS pharyngitis or persistent colonisation despite initial treatment. Factors contributing to antibiotic failure in the absence of antimicrobial resistance are poorly understood, but co-pathogenicity with beta-lactamase-producing flora, lack of compliance, the carrier state, poor antibiotic penetration into tonsillar tissues and re-infections may contribute [81,82]. Our post-antibiotic results are in line with another meta-analysis from 2004 that looked at bacteriological eradication after therapy for GAS pharyngotonsillitis [83]. A minority of studies (n = 13) in our review performed typing to distinguish between relapse and reacquisition with the original GAS strain and acquisition of a GAS new strain, however, the latter appears to be far less common. This may be an important consideration during prolonged outbreaks.

While most studies in our review reported use of standard bacitracin sensitivity and Lancefield grouping, the identification method was not reported in 10 studies. Furthermore, some studies used uncommon typing methods. Molecular diagnostic techniques are increasingly used in clinical microbiology and, while these approaches are more sensitive, it is not known whether a positive molecular diagnostic result carries the same public health risk as culture [84]. Future studies need to examine positivity based on molecular, point-of-care and culture testing.

Further research is needed to quantify clearance rates at distinct periods within the first 24 h of treatment and to determine whether non-beta-lactam antibiotics are effective, particularly when macrolide resistance is present or suspected and in patients who are asymptomatic or at high risk of severe GAS infection. Furthermore, research to better understand why one in 10 people either do not clear or reacquire GAS carriage after treatment would be invaluable. Other key research gaps include the optimal duration of antibiotics for eradication of GAS, the duration of infectivity following untreated symptomatic infection and the effectiveness of antibiotics in clearance of GAS from other body sites.

## Conclusion

Recent UK experience highlights the importance of a public health strategy of prompt isolation and treatment to reduce the incidence of iGAS through an overall reduction in circulating GAS infection. Our review provides evidence that antibiotics for pharyngeal GAS achieve a high rate of culture conversion within 24 h of starting antibiotic therapy. However, 10% of patients were culture-positive after treatment, predominantly with the original strain. Given the high secondary attack rate for GAS and the need to prevent onward transmission, this study provides evidence to support the public health guidelines that recommend that individuals with GAS pharyngitis or scarlet fever isolate from work or school for at least 24 h after starting antibiotic treatment for pharyngeal GAS. Our findings also support the approach of repeating throat swabs for individuals considered to be potential reservoirs of infection in invasive group A streptococcal infection outbreaks, as recurrence or relapse may occur soon after treatment completion.

## Supplementary Data

2262000 Supplement
