# Erratum for Euro Surveill. 2023;28(15)

**DOI:** 10.2807/1560-7917.ES.2023.28.17.230427e

**Published:** 2023-04-27

**Authors:** Eurosurveillance editorial team

**Affiliations:** 1 European Centre for Disease Prevention and Control (ECDC), Stockholm, Sweden

A correction was made to the article Time to negative throat culture following initiation of antibiotics for pharyngeal group A Streptococcus: a systematic review and meta-analysis up to October 2021 to inform public health control measures by McGuire et al. published on 13 April 2023. In the originally published version of this article, oxazolidinones was misspelled. This was corrected on 21 April 2023, and we apologise for any inconvenience this error may have caused.

