# Supplementary material for: Time to negative throat culture following initiation of antibiotics for pharyngeal group A Streptococcus: a systematic review and meta-analysis up to October 2021 to inform public health control measures
Source: Euro Surveill. 2023 Apr 13;28(15):2200573. doi: 10.2807/1560-7917.ES.2023.28.15.2200573 (PMC10103550; doi:10.2807/1560-7917.ES.2023.28.15.2200573)
Supplement: Supplement [file 22-00573_MCGUIRE_SUPPLEMENT.pdf]

This supplementary material is hosted by Eurosurveillance as supporting information alongside the article 'Time to negative throat culture following initiation of antibiotics for pharyngeal group A *Streptococcus*: a systematic review and meta-analysis to inform public health control measures' on behalf of the authors who remain responsible for the accuracy and appropriateness of the content. The same standards for ethics, copyright, attributions and permissions as for the article apply. Supplements are not edited by Eurosurveillance and the journal is not responsible for the maintenance of any links or email addresses provided therein.

Contents

Figure S1. Proportion of patients with culture-confirmed group A streptococcal throat infection or carriage at day 1, day 2 and days 3-9 after starting penicillin V treatment (n=28 studies). 3

Figure S2. Meta-regression of proportion of patients with culture-confirmed group A streptococcal throat infection or carriage on days 1 to 9 after the start of any type of antibiotic therapy (n=42 studies). 4

Figure S3. Meta-regression of proportion of patients with culture-confirmed group A streptococcal throat infection or carriage on days 1 to 9 after the start of penicillins (n=34 studies). 5

Figure S4. Meta-regression of proportion of patients with culture-confirmed group A streptococcal throat infection or carriage on days 1 to 9 after the start of cephalosporins (n=14 studies). 6

Figure S5. Meta-regression of proportion of patients with culture-confirmed group A streptococcal throat infection or carriage on days 1 to 9 after the start of penicillin V (n=28 studies). 7

Figure S6. Proportion of patients with culture-confirmed group A streptococcal throat infection or carriage at different times after completing antibiotic treatment [a] (n=23 studies). 8

Figure S7. Proportion of patients with relapse or reacquisition of the original group A Streptococcal strain after completion of therapy by antibiotic class, where typing was reported (n=13 studies). 9

Figure S8. Proportion of patients with acquisition of a new strain of group A Streptococcal (GAS) after completion of therapy by antibiotic class, where typing was reported (n=13 studies). 10

Figure S9. Proportion of patients who reported any side effect or adverse drug reaction by antibiotic class, where reported (n=14 studies). 11

Figure S10. Proportion of patients who ceased the study drug due to a side effect or adverse drug reaction by antibiotic class, where reported (n=12 studies). 12

Supplementary Appendix A. Search strategy 13
Supplementary Appendix B. Inclusion and exclusion criteria 17
Supplementary Appendix C. Risk of bias assessments of included studies 18
Supplementary Appendix D. Proportion of patients with culture-confirmed group A streptococcal throat carriage in studies reporting on macrolides, lincosamides and sulphonamides (n=15). 20
Supplementary Appendix E. Evidence of heterogeneity and of differences between sub-groups 21
Supplementary Appendix F. Sensitivity analyses excluding Brook *et al* outlier. 22
Supplementary Appendix G. Sensitivity analysis excluding studies that included participants with asymptomatic carriage (Edmond *et al*, Hoskins *et al* and Howie *et al*). 23
References for supplementary material 24

Figure S1. Proportion of patients with culture-confirmed group A streptococcal throat infection or carriage at day 1, day 2 and days 3-9 after starting penicillin V (n=28 studies).

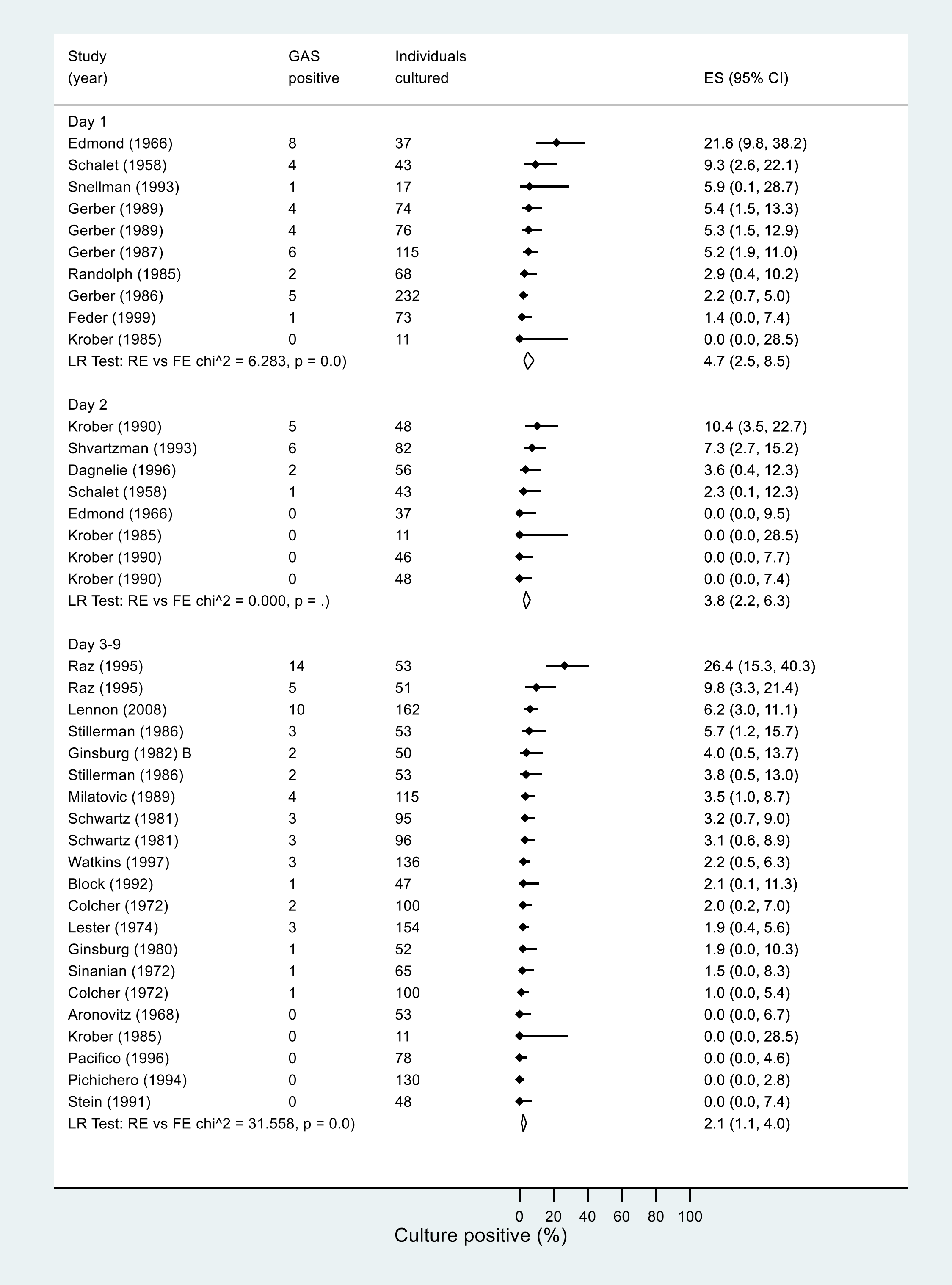

CI: confidence interval; ES: effect size; FE: fixed effects; GAS: group A *Streptococcus*; LR: likelihood ratio; RE: random effects.

For each included study, the proportion of individuals who were culture positive (dots) and 95% confidence intervals (lines) are given. The Freeman-Tukey double arcsine transformation was used to calculate weighted pooled proportion estimates for each sub-group (diamond). Heterogeneity within subgroups was tested using the X^2 test and quantified by the I^2 statistic.

Figure S2. Meta-regression of proportion of patients with culture-confirmed group A streptococcal throat infection or carriage on days 1 to 9 after the start of any type of antibiotic therapy (n=42 studies).

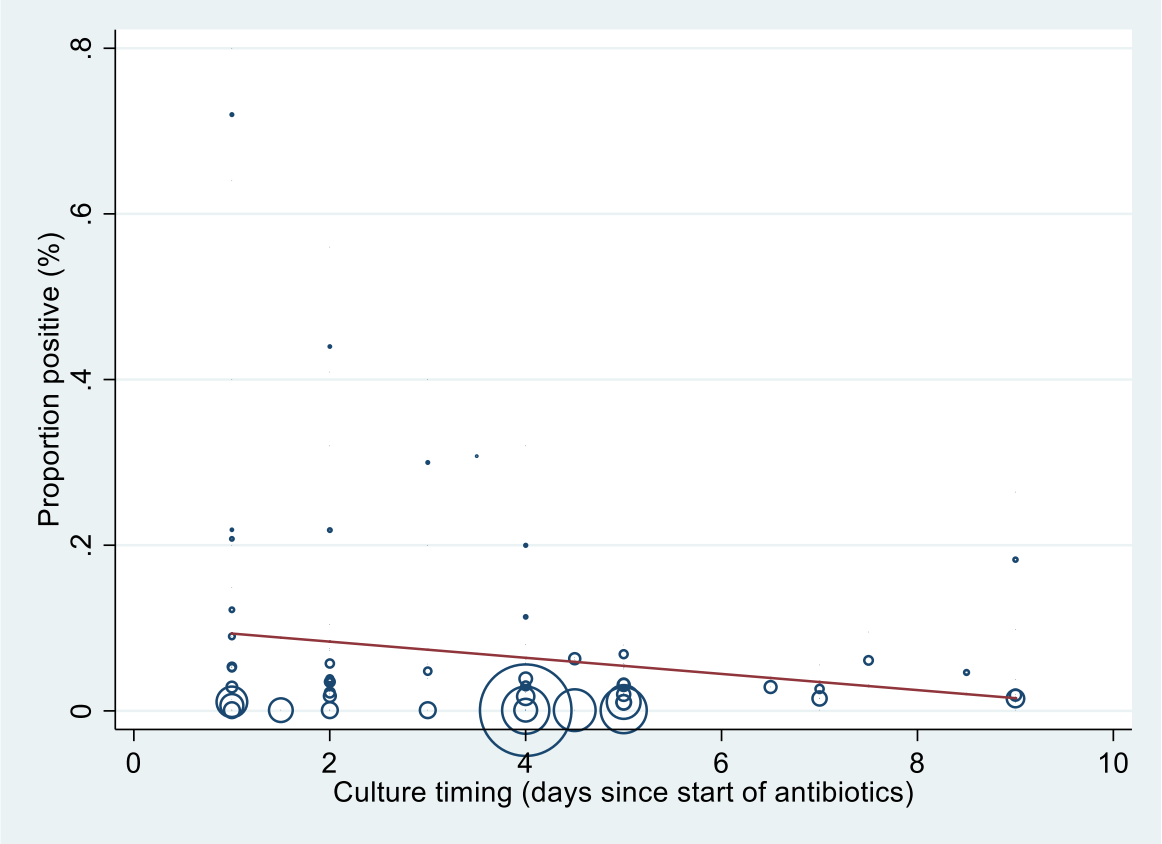

Figure S3. Meta-regression of proportion of patients with culture-confirmed group A streptococcal throat infection or carriage on days 1 to 9 after the start of penicillins (n=34 studies).

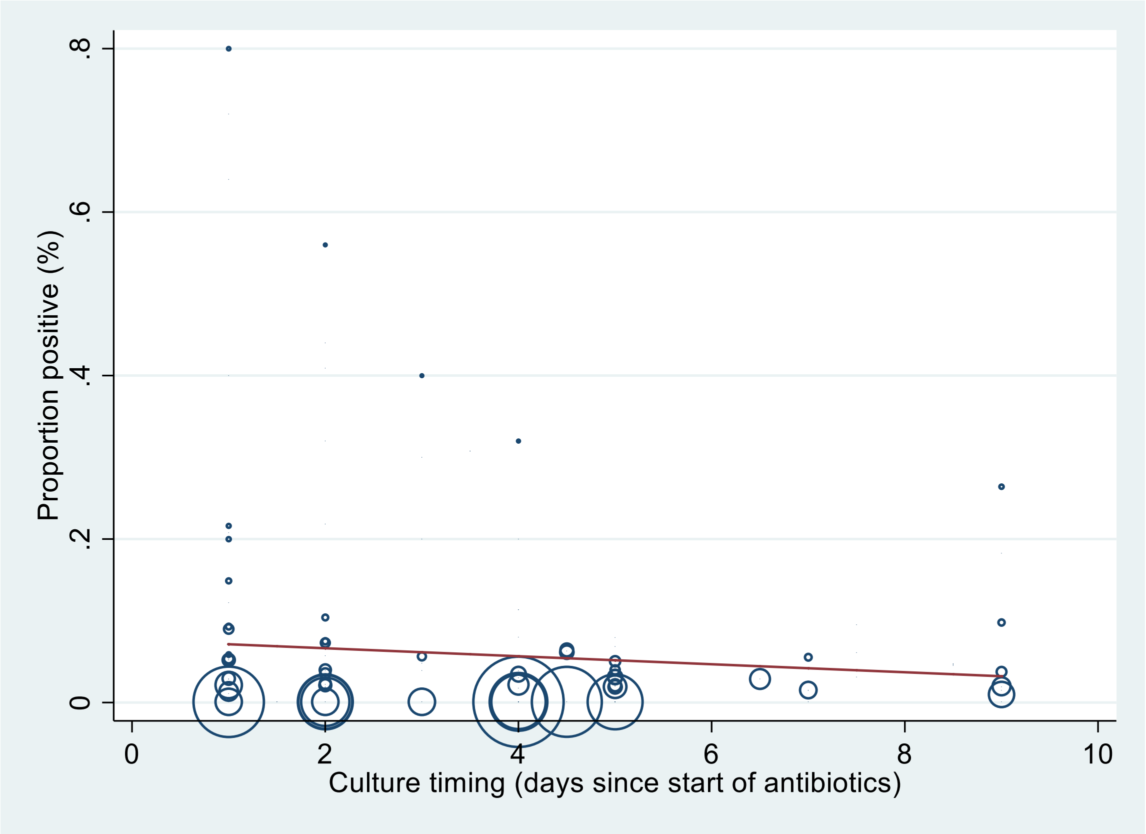

Figure S4. Meta-regression of proportion of patients with culture-confirmed group A streptococcal throat infection or carriage on days 1 to 9 after the start of cephalosporins (n=14 studies).

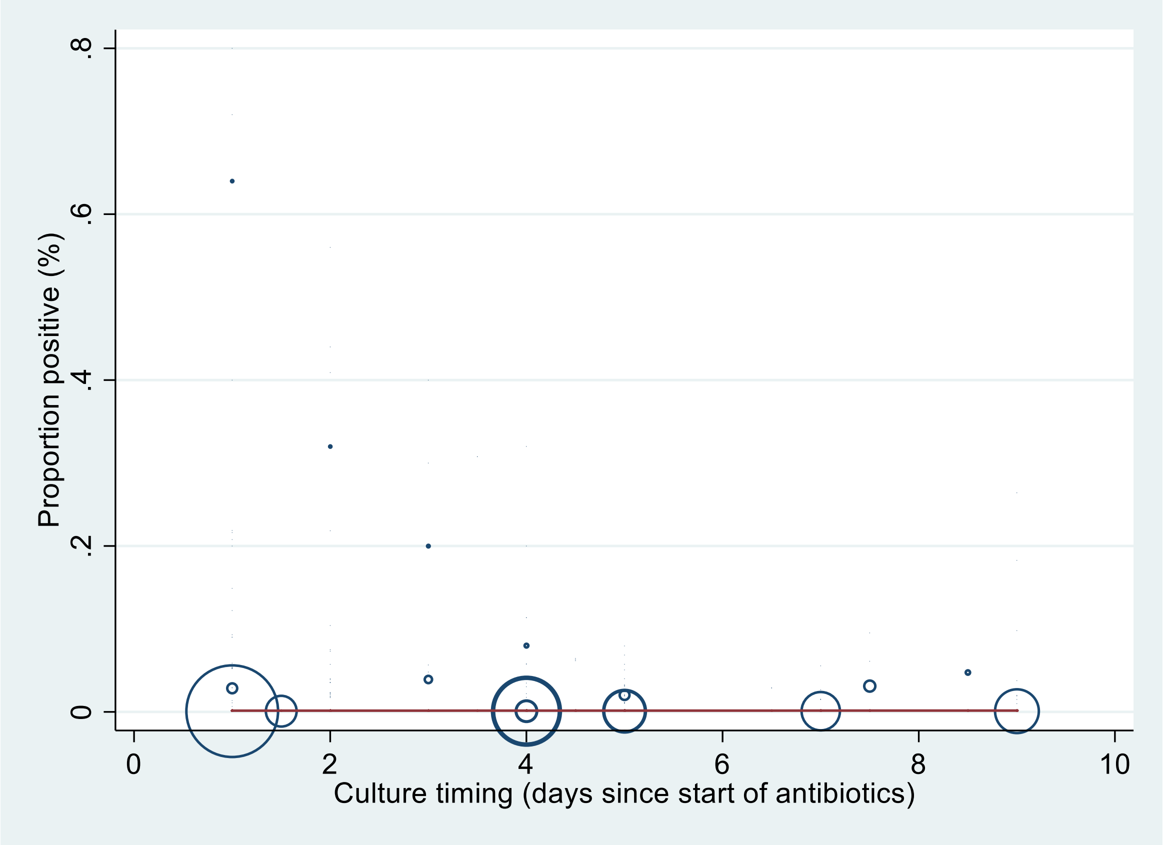

Figure S5. Meta-regression of proportion of patients with culture-confirmed group A streptococcal throat infection or carriage on days 1 to 9 after the start of penicillin V (n=28 studies).

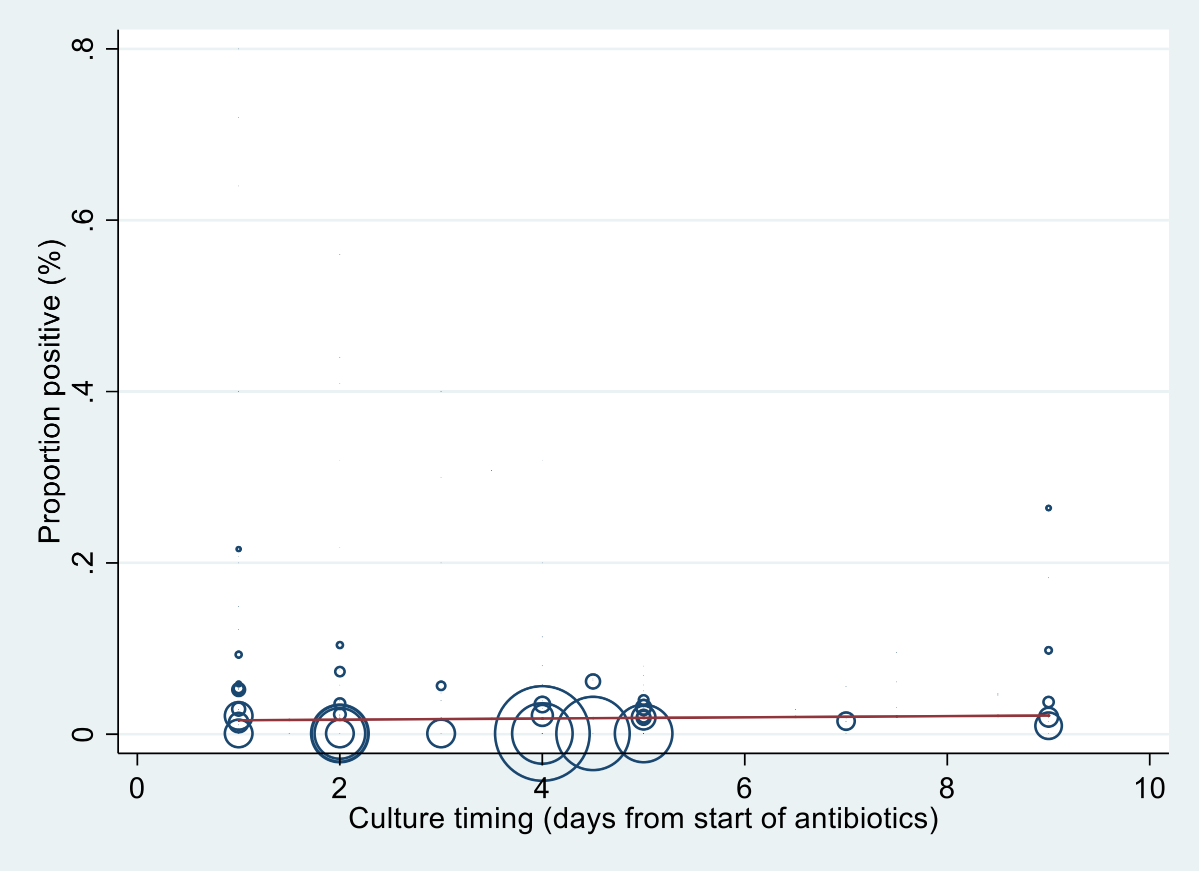

Figure S6. Proportion of patients with culture-confirmed group A streptococcal throat infection or carriage at different times after completing antibiotic treatment [a] (n=23 studies).

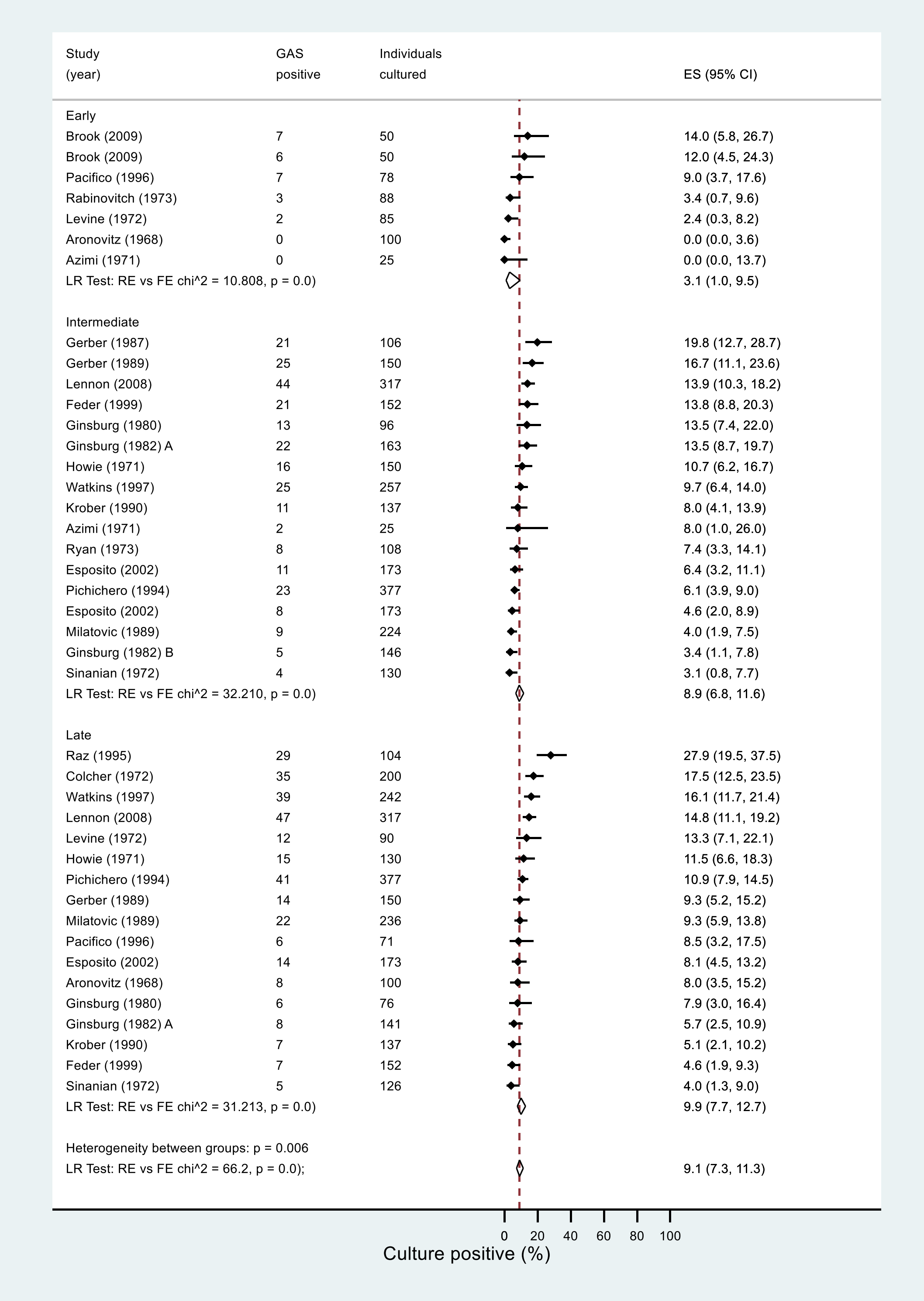

CI: confidence interval; ES: effect size; FE: fixed effects; GAS: group A *Streptococcus*; LR: likelihood ratio; RE: random effects.

For each included study, the proportion of individuals who were culture positive (dots) and 95% confidence intervals (lines) are given. The Freeman-Tukey double arcsine transformation was used to calculate weighted pooled proportion estimates for each sub-group (diamond). Heterogeneity within subgroups was tested using the X^2 test and quantified by the I^2 statistic.

[a] Early: <72hrs, Intermediate: 72hrs-10 days, Late: >10 days.

Figure S7. Proportion of patients with relapse or reacquisition of the original group A Streptococcal strain after completion of therapy by antibiotic class, where typing was reported (n=13 studies).

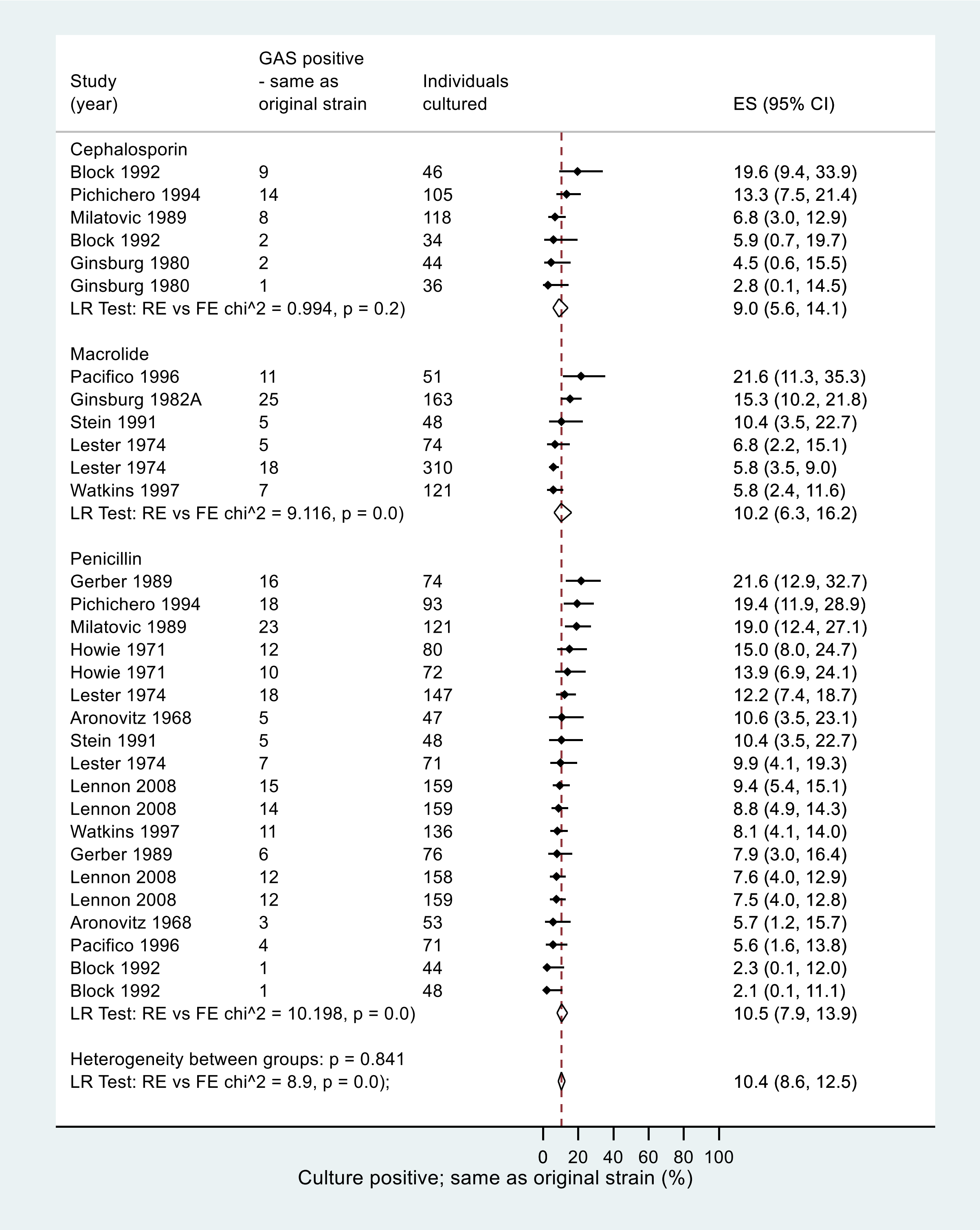

CI: confidence interval; ES: effect size; FE: fixed effects; GAS: group A *Streptococcus*; LR: likelihood ratio; RE: random effects.

For each included study, the proportion of individuals who were culture positive (dots) and 95% confidence intervals (lines) are given. The Freeman-Tukey double arcsine transformation was used to calculate weighted pooled proportion estimates for each sub-group (diamond). Heterogeneity within subgroups was tested using the X^2 test and quantified by the I^2 statistic.

Figure S8. Proportion of patients with acquisition of a new strain of group A Streptococcal (GAS) after completion of therapy by antibiotic class, where typing was reported (n=13 studies).

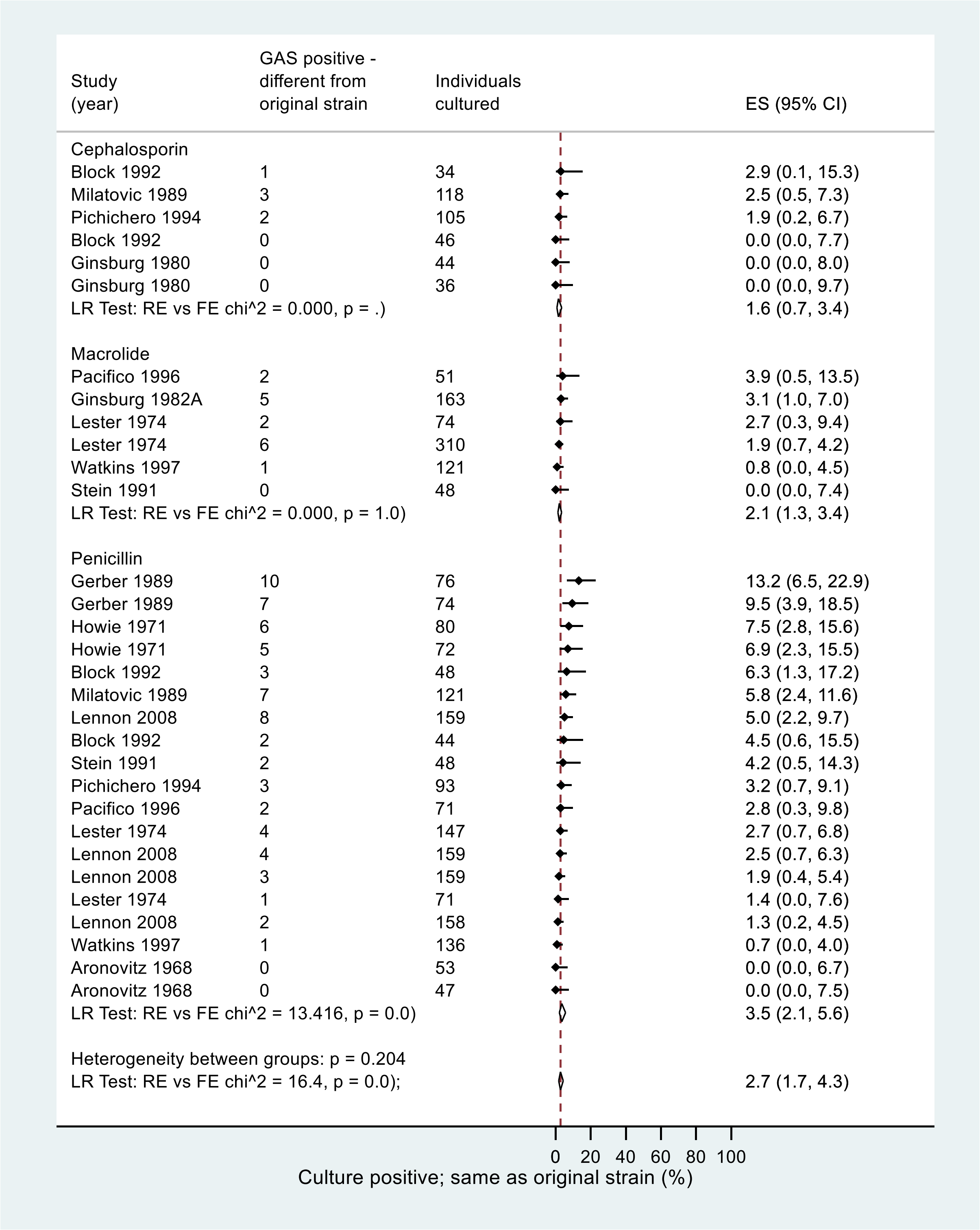

CI: confidence interval; ES: effect size; FE: fixed effects; GAS: group A *Streptococcus*; LR: likelihood ratio; RE: random effects.

For each included study, the proportion of individuals who were culture positive (dots) and 95% confidence intervals (lines) are given. The Freeman-Tukey double arcsine transformation was used to calculate weighted pooled proportion estimates for each sub-group (diamond). Heterogeneity within subgroups was tested using the X^2 test and quantified by the I^2 statistic.

Figure S9. Proportion of patients who reported any side effect or adverse drug reaction by antibiotic class, where reported (n=14 studies).

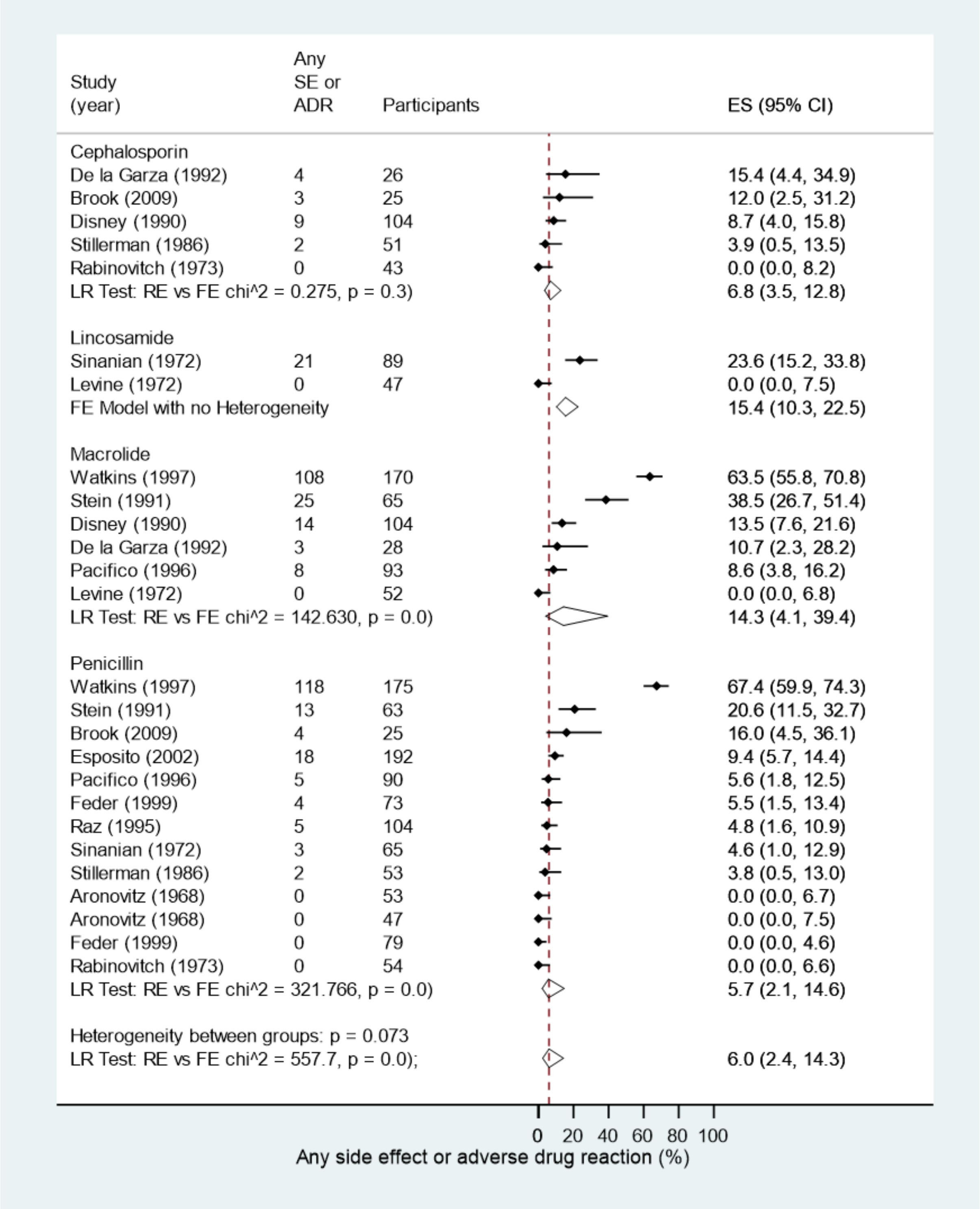

CI: confidence interval; ES: effect size; FE: fixed effects; GAS: group A *Streptococcus*; LR: likelihood ratio; RE: random effects.

For each included study, the proportion of individuals who were culture positive (dots) and 95% confidence intervals (lines) are given. The Freeman-Tukey double arcsine transformation was used to calculate weighted pooled proportion estimates for each sub-group (diamond). Heterogeneity within subgroups was tested using the X^2 test and quantified by the I^2 statistic.

Figure S10. Proportion of patients who ceased the study drug due to a side effect or adverse drug reaction by antibiotic class, where reported (n=12 studies).

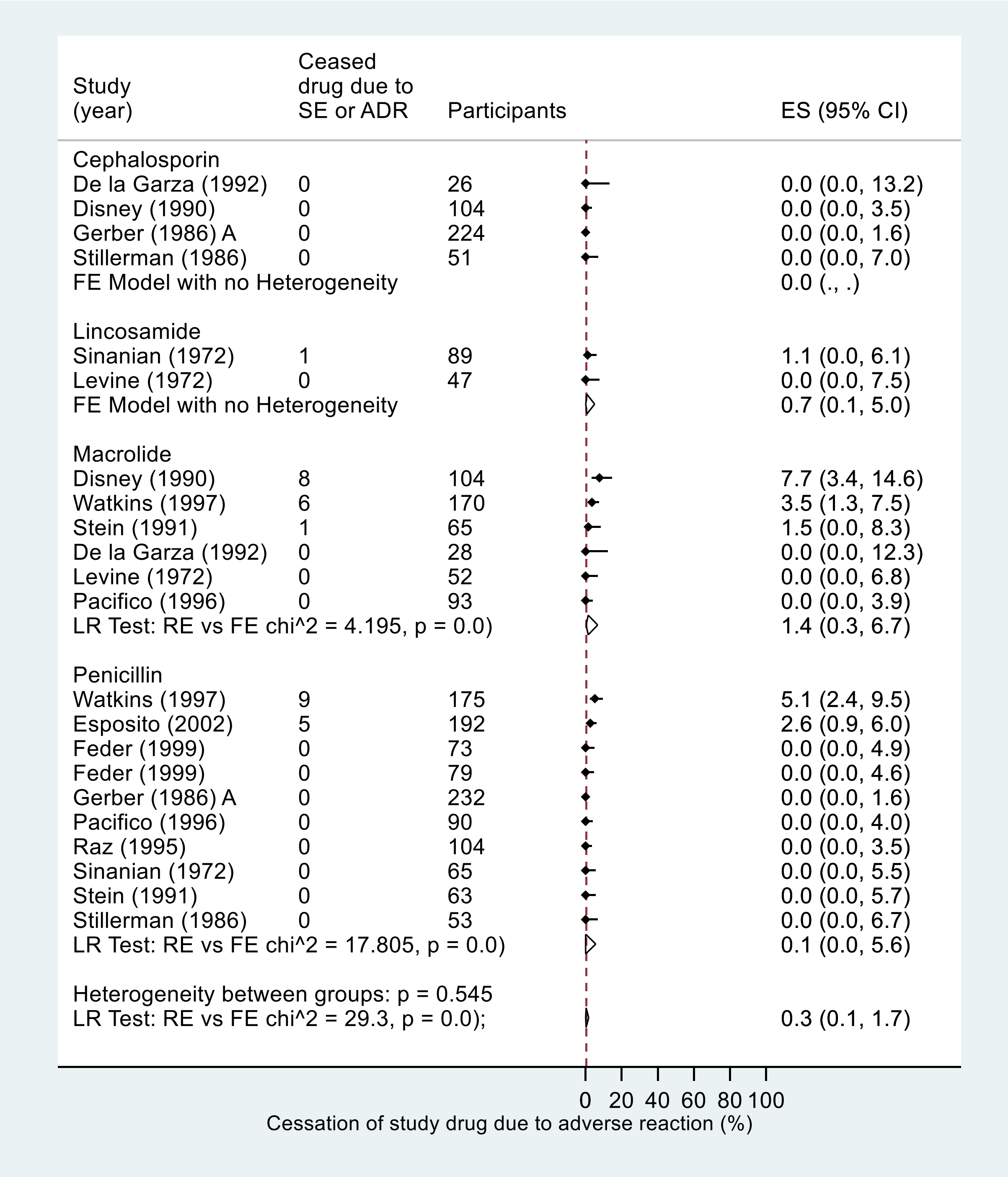

CI: confidence interval; ES: effect size; FE: fixed effects; GAS: group A *Streptococcus*; LR: likelihood ratio; RE: random effects.

For each included study, the proportion of individuals who were culture positive (dots) and 95% confidence intervals (lines) are given. The Freeman-Tukey double arcsine transformation was used to calculate weighted pooled proportion estimates for each sub-group (diamond). Heterogeneity within subgroups was tested using the X^2 test and quantified by the I^2 statistic.

Supplementary Appendix A. Search strategy

Database: Ovid MEDLINE(R) ALL <1946 to October 18, 2021>
Search Strategy:
----
1 streptococcal infections/ or rheumatic fever/ or scarlet fever/ (45136)
2 "streptococc* infection* ".tw,kw. (6645)
3 "scarlet fever".kw,tw. (2367)
4 pharyngitis.kw,tw. (6051)
5 pyogenes.kw,tw. (10230)
6 (strep* adj2 Carriage).kw,tw. (526)
7 or/1-6 (58878)
8 exp Anti-Bacterial Agents/ (764976)
9 exp Penicillins/ (82399)
10 Azithromycin/ (6120)
11 Cefaclor/ (841)
12 Cefadroxil/ (417)
13 cefdinir/ (252)
14 Cefixime/ (794)
15 Cefotiam/ (414)
16 Ceftizoxime/ (1148)
17 Ceftibuten/ (215)
18 Cefuroxime/ (2246)
19 exp Cephalosporins/ (44226)
20 Clarithromycin/ (6572)
21 clindamycin/ (5882)
22 erythromycin/ (13955)
23 josamycin/ (235)
24 lincosamide/ (465)
25 loracarbef/ (0)
26 macrolide/ (13124)
27 penicillin derivative/ (0)
28 penicillin V/ (2180)
29 roxithromycin/ (836)
30 spiramycin/ (725)
31 telithromycin/ (0)
32 Amoxicillin/ (9913)
33 Amoxicillin-Potassium Clavulanate Combination/ (2674)
34 Clavulanic Acid/ (1678)
35 Quinolones/ (12329)
36 Moxifloxacin/ (2692)
37 Gemifloxacin/ (278)
38 Levofloxacin/ (3629)
39 (azithromycin or cefaclor or cefadroxil or cefcapene or cefdinir or cefixime or cefotiam or cefpodoxime or ceftibuten or cefuroxime or cephalosporin or clarithromycin or "clavulanic acid" or clindamycin or erythromycin or josamycin or lincosamide or loracarbef or macrolide or penicillin or roxithromycin or spiramycin or telithromycin).kw,tw. (119821)
40 (Amoxicillin or Augmentin or Co-amoxiclav or Clavulanate or Quinolone or Moxifloxacin or Gemifloxacin or Levofloxacin).kw,tw. (38019)
41 exp Antibiotic Prophylaxis/ (14723)
42 Chemoprevention/ (6331)
43 (Prophylaxis or Chemoprophylaxis).kw,tw. (107937)
44 or/8-43 (911843)
45 clinical effectiveness/ (1060749)
46 intervention study/ (531530)
47 time factor/ (1217374)
48 outcome assessment/ (0)
49 Outcome Assessment, Health Care/ (77921)

50 treatment outcome/ (1060749)
51 "eradicat*".kw,tw. (68818)
52 Clearance.kw,tw. (172556)
53 or/45-52 (2815855)
54 7 and 44 and 53 (2779)
55 limit 54 to english language (2216)
56 limit 55 to humans (1998)
57 (letter or historical article or comment or editorial or news).pt. (2537403)
58 56 not 57 (1910)

****

Database: Embase <1974 to 2021 October 25>
Search Strategy:
----
1 exp streptococcus infection/ or exp group a streptococcal infection/ (51972)
2 exp rheumatic fever/ (7775)
3 exp scarlet fever/ (1453)
4 "streptococc* infection*".kw,tw. (5851)
5 "scarlet fever".kw,tw. (1078)
6 pharyngitis.kw,tw. (7924)
7 pyogenes.kw,tw. (11636)
8 (strep* adj2 Carriage).kw,tw. (624)
9 or/1-8 (69197)
10 exp antiinfective agent/ (3798679)
11 exp penicillin derivative/ (313240)
12 azithromycin/ (43793)
13 cefaclor/ (8195)
14 cefadroxil/ (3657)
15 cefdinir/ (2386)
16 cefixime/ (8447)
17 cefotiam/ (3186)
18 ceftizoxime/ (4107)
19 ceftibuten/ (1381)
20 cefuroxime axetil/ or cefuroxime/ (27696)
21 exp cephalosporin derivative/ (247374)
22 amoxicillin plus clarithromycin plus omeprazole/ or clarithromycin/ or exp clarithromycin derivative/ or amoxicillin plus clarithromycin plus lansoprazole/ (39249)
23 clindamycin/ (54805)
24 erythromycin/ or exp erythromycin derivative/ (75866)
25 josamycin/ (2308)
26 lincosamide/ (2997)
27 loracarbef/ (1072)
28 macrolide/ (33829)
29 roxithromycin/ (5738)
30 spiramycin/ (4448)
31 telithromycin/ (2942)
32 amoxicillin plus clavulanic acid/ or amoxicillin/ or exp amoxicillin derivative/ (98808)
33 clavulanic acid/ (14642)
34 exp quinolone derivative/ (186769)
35 moxifloxacin/ (20882)
36 gemifloxacin/ (1589)
37 levofloxacin/ (41168)
38 (azithromycin or cefaclor or cefadroxil or cefcapene or cefdinir or cefixime or cefotiam or cefpodoxime or ceftibuten or cefuroxime or cephalosporin or clarithromycin or "clavulanic acid" or clindamycin or erythromycin or josamycin or lincosamide or loracarbef or macrolide or penicillin or roxithromycin or spiramycin or telithromycin).kw,tw. (139399)

39 (Amoxicillin or Augmentin or Co-amoxiclav or Clavulanate or Quinolone or Moxifloxacin or Gemifloxacin or Levofloxacin).kw,tw. (59308)
40 Antibiotic Prophylaxis.kw,tw. (15251)
41 exp antibiotic prophylaxis/ (34332)
42 exp chemoprophylaxis/ (26282)
43 (Prophylaxis or Chemoprophylaxis).kw,tw. (163016)
44 or/10-43 (3947417)
45 exp comparative effectiveness/ or exp clinical effectiveness/ (255315)
46 exp intervention study/ (52425)
47 exp time factor/ (42129)
48 exp outcome assessment/ (629063)
49 exp treatment outcome/ (1864693)
50 outcome.ti. (240561)
51 "eradicat*".kw,tw. (91853)
52 Clearance.kw,tw. (235348)
53 or/45-52 (2529184)
54 9 and 44 and 53 (4030)
55 (54 and english.lg.) not (letter or editorial).pt. not (nonhuman/ not human/) not (conference abstract or conference paper or conference proceeding or "conference review").pt. (2808)

****

Search Name: 20211101MCPenicillinTreatmentStreptococcalInfections58989
Date Run: 26/10/2021 14:51:04
Comment:

ID	Search	Hits
#1	MeSH descriptor: [Streptococcal Infections] explode all trees	1533
#2	MeSH descriptor: [Rheumatic Fever] explode all trees	188
#3	MeSH descriptor: [Scarlet Fever] explode all trees	12
#4	(streptococc* AND infection*):ti,ab,kw	2919
#5	("scarlet fever"):ti,ab,kw	55
#6	(pharyngitis):ti,ab,kw	2468
#7	(pyogenes):ti,ab,kw	502
#8	(strep* AND Carriage):ti,ab,kw	243
#9	#1 OR #2 OR #3 OR #4 OR #5 OR #6 OR #7 OR #8	5729
#10	MeSH descriptor: [Anti-Bacterial Agents] explode all trees	12590
#11	MeSH descriptor: [Penicillins] explode all trees	5783
#12	MeSH descriptor: [Azithromycin] explode all trees	1052
#13	MeSH descriptor: [Cefaclor] explode all trees	230
#14	MeSH descriptor: [Cefadroxil] explode all trees	94
#15	MeSH descriptor: [Cefdinir] explode all trees	64
#16	MeSH descriptor: [Cefixime] explode all trees	145
#17	MeSH descriptor: [Cefotiam] explode all trees	46
#18	MeSH descriptor: [Ceftizoxime] explode all trees	174
#19	MeSH descriptor: [Ceftibuten] explode all trees	42
#20	MeSH descriptor: [Cefuroxime] explode all trees	468
#21	MeSH descriptor: [Cephalosporins] explode all trees	4439
#22	MeSH descriptor: [Clarithromycin] explode all trees	1510
#23	MeSH descriptor: [Clindamycin] explode all trees	893
#24	MeSH descriptor: [Erythromycin] explode all trees	3485
#25	MeSH descriptor: [Josamycin] explode all trees	21
#26	MeSH descriptor: [Lincosamides] explode all trees	935
#27	MeSH descriptor: [Macrolides] explode all trees	9459
#28	MeSH descriptor: [Roxithromycin] explode all trees	120
#29	MeSH descriptor: [Spiramycin] explode all trees	29
#30	MeSH descriptor: [Amoxicillin] explode all trees	2914

#31 MeSH descriptor: [Clavulanic Acids] explode all trees 900
#32 MeSH descriptor: [Quinolones] explode all trees 5162
#33 MeSH descriptor: [Moxifloxacin] explode all trees882
#34 MeSH descriptor: [Gemifloxacin] explode all trees 47
#35 MeSH descriptor: [Levofloxacin] explode all trees 655
#36 (azithromycin or cefaclor or cefadroxil or cefcapene or cefdinir or cefixime or cefotiam or cefpodoxime or ceftibuten or cefuroxime or cephalosporin or clarithromycin or clavulanic acid or clindamycin or erythromycin or josamycin or lincosamide or loracarbef or macrolide or penicillin or roxithromycin or spiramycin or telithromycin):ti,ab,kw 15873
#37 (Amoxicillin or Augmentin or Co-amoxiclav or Clavulanate or Quinolone or Moxifloxacin or Gemifloxacin or Levofloxacin):ti,ab,kw 9426
#38 MeSH descriptor: [Antibiotic Prophylaxis] explode all trees 1315
#39 (Prophylaxis or Chemoprophylaxis):ti,ab,kw 27481
#40 #10 or #11 or #12 or #13 or #14 or #15 or #16 or #17 or #18 or #19 or #20 or #21 or #22 or #23 or #24 or #25 or #26 or #27 or #28 or #29 or #30 or #31 or #32 or #33 or #34 or #35 or #36 or #37 or #38 or #39 63223
#41 #9 AND #40 2107
#42 MeSH descriptor: [Treatment Outcome] explode all trees 146733
#43 (outcome or intervention or effective* or eridcat* or clearance):ti,ab,kw 870925
#44 #42 or #43 874825
#45 #41 and #44 1200

Supplementary Appendix B. Inclusion and exclusion criteria

	Inclusion criteria	Exclusion criteria
**Population**	• Culture confirmed GAS pharyngitis or scarlet fever or with asymptomatic pharyngeal GAS carriage†	• Studies which did not report on participants with pharyngeal GAS (including those with impetigo, erysipelas, or iGAS e.g. necrotising fasciitis, pneumonia, and bacteraemia) unless a sub-group with culture confirmed pharyngeal GAS were also reported.<br>• Studies which only reported treatment outcomes in patients with recurrent GAS pharyngitis (as we considered this to be a sub-population which may have issues affecting treatment efficacy such as penicillin tolerance, compliance issues or beta-lactamase producing co-pathogens).
**Intervention**	• Any antibiotic	• Studies which only reported on the use of herbal medicines or probiotics.
**Outcome**	• Rates of positive or negative GAS throat culture at defined time points during antibiotics or time to clearance (mean or median) after the initiation of antibiotics	• Studies which only reported bacteriological outcomes after the completion of antibiotic treatment<br>• Studies that included the study population as a sub-group, but which do not present disaggregated data for the outcome measure.
**Study type**	• All peer reviewed primary research studies with 10 or more participants were considered regardless of design.	• Studies which only reported PCR or RADT (without throat culture) or which did not differentiate GAS from beta-haemolytic streptococci.<br>• Animal or in vitro studies.<br>• Case reports, letters, commentaries, and conference abstracts.<br>• Systematic reviews and meta-analyses were excluded but any reviews identified during the title and abstract screening were used to screen for further eligible studies.<br>• Duplicate publications of the same raw data.

GAS: group A *Streptococcus*; iGAS: invasive GAS infection; PCR: polymerase chain reaction; RADT: rapid antigen detection test

† Acceptable confirmation methods were those listed in the WHO publication "Laboratory diagnosis of group A streptococcal infections".[1] Studies which did not state method of laboratory confirmation but reported diagnosis of group A streptococcal infection (rather than unqualified 'Streptococcal infection' alone) were also included.

Supplementary Appendix C. Risk of bias assessments of included studies

NIHR Tool - Quality Assessment of Controlled Intervention Studies															
**Author year**	**Question**														**Risk of Bias**
	**1**	**2**	**3**	**4**	**5**	**6**	**7**	**8**	**9**	**10**	**11**	**12**	**13**	**14**	
Esposito 2002	Y	Y	Y	N	Y	Y	Y	Y	Y	Y	Y	NR	Y	Y	Low
Gerber 1986A	Y	CD	NR	NR	NR	NR	Y	Y	Y	NR	Y	NR	Y	CD	Moderate
Snellman 1993	Y	Y	N	N	N	NR	Y	CD	Y	Y	Y	NR	N	NR	High
Randolph 1985	Y	Y	Y	Y	Y	Y	Y	Y	Y	Y	Y	NR	Y	CD	Low
Gerber 1989	Y	CD	CD	NR	NR	N	Y	CD	N	NR	Y	NR	Y	N	High
Ginsburg 1982A	Y	CD	NR	NR	Y	Y	Y	Y	Y	NR	Y	NR	Y	N	Moderate
Lennon 2008	Y	Y	Y	N	NR	Y	Y	Y	Y	NR	Y	Y	Y	N	Low
Feder 1999	Y	Y	Y	N	Y	Y	Y	Y	N	NR	Y	NR	Y	N	Moderate
Krober 1990	Y	Y	NR	NR	NR	Y	Y	CD	CD	NR	Y	NR	Y	N	Moderate
Schwartz 1981	Y	CD	NR	N	NR	Y	Y	CD	CD	NR	Y	NR	Y	N	High
Schwartz 2015	Y	Y	NR	N	N	Y	Y	CD	Y	NR	Y	NR	Y	N	Moderate
Shvartzman 1993	Y	CD	NR	N	NR	Y	CD	CD	NR	NR	CD	NR	Y	N	High
Lester 1974	Y	Y	Y	N	NR	Y	Y	Y	CD	NR	Y	NR	Y	CD	Moderate
Trickett 1973	Y	CD	Y	Y	Y	Y	Y	CD	NR	Y	CD	NR	Y	N	Moderate
Pacifico 1996	Y	Y	NR	N	NR	Y	Y	Y	Y	CD	Y	NR	Y	N	Moderate
Stein 1991	Y	Y	NR	Y	NR	Y	Y	CD	NR	Y	Y	NR	Y	N	Moderate
Block 1992	Y	Y	NR	N	N	Y	Y	Y	Y	Y	Y	NR	Y	N	Moderate
Mogabgab 1976	N	N	NR	NR	NR	N	NR	NR	NR	NR	Y	NR	Y	Y	High
Levine 1972	Y	CD	NR	Y	Y	Y	Y	Y	NR	NR	Y	NR	Y	N	Moderate
Watkins 1997	Y	Y	Y	Y	Y	Y	Y	Y	NR	NR	Y	NR	Y	N	Low
Edmond 1966	N	NR	NR	Y	Y	NR	CD	CD	NR	NR	Y	NR	Y	NR	High
Disney 1990	Y	CD	NR	N	N	Y	Y	CD	Y	Y	Y	NR	Y	N	Moderate
Stillerman 1986	Y	Y	N	N	N	Y	Y	CD	Y	NR	Y	NR	Y	N	Moderate
Ginsburg 1980	Y	CD	NR	N	N	Y	Y	Y	CD	NR	Y	NR	Y	N	Moderate
Dagnelie 1996	Y	CD	NR	Y	Y	CD	Y	Y	NR	Y	Y	NR	Y	Y	Moderate
Pichichero 1994	Y	CD	NR	N	Y	Y	Y	CD	Y	Y	Y	NR	Y	N	Moderate
Ryan 1973	Y	CD	Y	NR	Y	NR	CD	CD	NR	Y	Y	NR	Y	CD	Moderate
Colcher 1972	Y	CD	NR	N	N	Y	Y	CD	N	NR	Y	NR	Y	N	High
Raz 1995	Y	CD	NR	N	N	Y	Y	Y	N	NR	Y	NR	Y	N	Moderate
Pavesio 1988	Y	CD	NR	N	N	Y	CD	CD	NR	NR	CD	NR	Y	CD	High
Sinanian 1972	Y	NR	CD	N	N	CD	Y	Y	NR	NR	CD	NR	N	Y	High
Ginsburg 1982B	Y	CD	NR	N	N	Y	NR	NR	Y	NR	Y	NR	Y	NR	High
Hoskins 1981	Y	CD	NR	N	N	NR	CD	CD	NR	NR	N	NR	Y	N	High
Krober 1985	Y	Y	Y	Y	Y	Y	Y	Y	Y	Y	Y	NR	Y	Y	Low
Aronovitz 1968	Y	Y	Y	Y	Y	CD	Y	Y	N	Y	Y	NR	Y	Y	Low
Schalet 1958	N	NR	NR	N	Y	CD	Y	Y	Y	Y	Y	NR	CD	NR	High
Howie 1971	Y	Y	NR	N	N	CD	CD	CD	Y	Y	Y	NR	Y	CD	High

Rabinovitch 1973	Y	CD	NR	NR	NR	NR	N	CD	Y	NR	Y	NR	Y	N	High
Milatovic 1989	Y	CD	N	NR	NR	CD	Y	NR	CD	NR	Y	NR	Y	N	High
De La Garza 1992	Y	Y	NR	N	N	N	N	Y	Y	Y	Y	NR	Y	N	Moderate

Y: yes; N: no; CD: cannot determine; NR: not reported
Q1-14: questions 1 to 14 for each NIHR tool:[2]
Q1: Was the study described as randomized, a randomized trial, a randomized clinical trial, or an RCT?
Q2: Was the method of randomization adequate (i.e., use of randomly generated assignment)?
Q3: Was the treatment allocation concealed (so that assignments could not be predicted)?
Q4: Were study participants and providers blinded to treatment group assignment?
Q5: Were the people assessing the outcomes blinded to the participants' group assignments?
Q6: Were the groups similar at baseline on important characteristics that could affect outcomes (e.g., demographics, risk factors, co-morbid conditions)?
Q7: Was the overall drop-out rate from the study at endpoint 20% or lower of the number allocated to treatment?
Q8: Was the differential drop-out rate (between treatment groups) at endpoint 15 percentage points or lower?
Q9: Was there high adherence to the intervention protocols for each treatment group?
Q10: Were other interventions avoided or similar in the groups (e.g., similar background treatments)?
Q11: Were outcomes assessed using valid and reliable measures, implemented consistently across all study participants?
Q12: Did the authors report that the sample size was sufficiently large to be able to detect a difference in the main outcome between groups with at least 80% power?
Q13: Were outcomes reported or subgroups analysed prespecified (i.e., identified before analyses were conducted)?
Q14: Were all randomized participants analysed in the group to which they were originally assigned, i.e., did they use an intention-to-treat analysis?

**NIHR Tool – Quality Assessment of for Before-After (Pre-Post) Studies with No Control Group**													
**Author year**	**Question**												**Risk of Bias**
	**1**	**2**	**3**	**4**	**5**	**6**	**7**	**8**	**9**	**10**	**11**	**12**	
Brook 2009	Y	Y	Y	CD	NR	Y	Y	N	Y	N	N	NR	Moderate
Azimi 1971	Y	N	NR	CD	NR	Y	Y	N	N	N	N	NR	High
Gerber 1987	Y	N	CD	CD	NR	N	Y	N	N	N	N	NR	High

Y: yes; N: no; CD: cannot determine; NR: not reported
Q1-12: questions 1 to 14 for each NIHR tool:[2]
Q1: Was the study question or objective clearly stated?
Q2: Were eligibility/selection criteria for the study population prespecified and clearly described?
Q3: Were the participants in the study representative of those who would be eligible for the test/service/intervention in the general or clinical population of interest?
Q4: Were all eligible participants that met the prespecified entry criteria enrolled?
Q5: Was the sample size sufficiently large to provide confidence in the findings?
Q6: Was the test/service/intervention clearly described and delivered consistently across the study population?
Q7: Were the outcome measures prespecified, clearly defined, valid, reliable, and assessed consistently across all study participants?
Q8: Were the people assessing the outcomes blinded to the participants' exposures/interventions?
Q9: Was the loss to follow-up after baseline 20% or less? Were those lost to follow-up accounted for in the analysis?
Q10: Did the statistical methods examine changes in outcome measures from before to after the intervention? Were statistical tests done that provided p values for the pre-to-post changes?
Q11: Were outcome measures of interest taken multiple times before the intervention and multiple times after the intervention (i.e., did they use an interrupted time-series design)?
Q12: If the intervention was conducted at a group level (e.g., a whole hospital, a community, etc.) did the statistical analysis take into account the use of individual-level data to determine effects at the group level?

Supplementary Appendix D. Proportion of patients with culture-confirmed group A streptococcal throat carriage in studies reporting on macrolides, lincosamides and sulphonamides (n=15).

Study (Reference)	Antibiotic regimen	Day of culture	Proportion culture-positive
**Macrolides**			
Snellman 1993 (3)	Erythromycin 250mg TDS	Day 1	6/15 (40.0%)
Ryan 1973 (4)	Erythromycin 30-50mg/kg/day QDS	Day 1-3	2/110 (1.8%)
Levine 1972 (5)	Erythromycin 16mg/kg/day TDS/QDS	Day 2-6	3/52 (5.8%)
Watkins 1997 (6)	Dirithromycin 500mg OD	Day 3-5	7/121 (5.8%)
Stein 1991 (7)	Clarithromycin 250mg BD	Day 4-6	0/47 (0%)
Ginsburg 1982A (8)	Erythromycin 15mg/kg/day BD	Day 5	12/175 (6.9%)
Lester 1974 (9)	Erythromycin 500mg/day if <22.6kg, 1g/day if >22.7kg QDS	Day 5	2/74 (2.7%)
Ginsburg 1982B (10)	Erythromycin 15mg/kg/day BD	Day 5	0/50 (0%)
Disney 1990 (11)	Erythromycin 30mg/kg/day QDS	Day 7-8	8/84 (9.5%)
De la Garza 1992 (12)	Erythromycin 30mg/kg/day OD	Day 7-10	1/22 (4.5%)
**Lincosamides**			
Levine 1972 (5)	Clindamycin 16mg/kg/day TDS/QDS	Day 2-6	0/47 (0%)
Lester 1974 (9)	Clindamycin palmitate <24.9kg 300mg/day QDS, 25-34kg 450mg/day TDS, >34.1 600mg/day QDS, Clindamycin HCL <24.9kg 300mg/day QDS, 25-34kg 450mg/day TDS, >34.1 600mg/day QDS or Clindamycin HCL <24.9kg 300mg/day >25kg 600mg/day BD	Day 5	1/323 (0.3%)
Sinanian 1972 (13)	Clindamycin Up to 55lb 75mg QDS to 150mg TDS, 55lb-75lb 150mg TDS to 150mg QDS, >75lb 150mg QDS to 300mg QDS	Day 7	1/67 (1.5%)
**Sulphonamides**			
Trickett 1973 (14)	Co-trimoxazole 2 tablets BD (80/400)	Day 2	18/44 (40.9%)
Hoskins 1981 (15)	Co-trimoxazole 2 tablets BD (125/375)	Day 3-4	4/13 (30.8%)

Supplementary Appendix E. Evidence of heterogeneity and of differences between sub-groups

	LR test statistic	Degrees of Freedom	p-value	Tau^2
**Figure 2**				
Day 1	180.0	9	<0.01	2.6
Day 2	58.3	7	<0.01	1.7
Day 3-9	120.2	29	<0.01	1.4
Test for heterogeneity between sub-groups	2.7	2	0.3	-
**Figure 3**				
Day 1	95.8	13	<0.01	2.5
Day 2	33.8	11	<0.01	1.7
Day 3-9	74.9	26	<0.01	1.4
Test for heterogeneity between sub-groups	2.0	2	0.4	-
**Figure 4**				
Cephalosporin	1.1	4	0.1	0.1
Macrolide	10.2	4	<0.01	0.3
Penicillin	26.0	17	<0.01	0.2
Overall	29.7	29	<0.01	0.2
Test for heterogeneity between sub-groups	1.6	2	0.5	-
**Figure S1**				
Day 1	-	1	-	15.3
Day 2	-	1	-	<0.01
Day 3-9	16.0	13	<0.01	2.8
Test for heterogeneity between sub-groups	8.6	2	<0.01	-
**Figure S2**				
Day 1	6.3	8	<0.01	0.5
Day 2	0.0	6	-	<0.01
Day 3-9	31.6	19	<0.01	0.9
Test for heterogeneity between sub-groups	3.5	2	0.2	-
**Figure S7**				
Early	10.8	5	<0.01	1.3
Intermediate	32.2	15	<0.01	0.3
Late	31.2	15	<0.01	0.2
Overall	66.2	39	<0.01	0.3
Test for heterogeneity between sub-groups	10.3	2	<0.01	-
**Figure S8**				
Cephalosporin	1.0	4	0.2	0.1
Macrolide	9.1	4	<0.01	0.3
Penicillin	10.2	17	<0.01	0.2
Overall	8.9	29	<0.01	0.1
Test for heterogeneity between sub-groups	0.3	2	0.8	-
**Figure S9**				
Cephalosporin	0.0	4	-	<0.01
Macrolide	0.0	4	1.0	<0.01
Penicillin	13.4	17	<0.01	0.4
Overall	16.4	29	<0.01	0.5
Test for heterogeneity between sub-groups	3.2	2	0.2	-
**Figure S10**				
Cephalosporin	0.3	3	0.3	0.2
Lincosamide	-	1	0	<0.01
Macrolide	142.6	4	<0.01	2.6
Penicillin	321.6	11	<0.01	2.7
Overall	557.7	24	<0.01	3.0
Test for heterogeneity between sub-groups	7.0	3	0.1	-
**Figure S11**				
Cephalosporin	-	2	-	1.0
Lincosamide	-	1	-	<0.01
Macrolide	4.2	4	<0.01	1.3
Penicillin	17.8	8	<0.01	10.1
Overall	29.3	20	<0.01	3.6
Test for heterogeneity between sub-groups	2.1	3	0.5	-

Supplementary Appendix F. Sensitivity analyses excluding Brook *et al* outlier.

	With Brook *et al*	Excluding Brook *et al*
**Overall**		
D1	6.9 (2.7-16.8)	5.2 (2.5-10.5)
D2	5.4 (2.1-13.3)	4.3 (2.0-8.9)
D3-9	2.6 (1.6-4.2)	2.4 (1.5-3.8)
**Penicillin**		
D1	6.5 (2.5-16.1)	5.0 (2.6-9.2)
D2	4.7 (1.7-12.4)	3.8 (2.5-5.7)
D3-9	2.6 (1.4-4.8)	2.4 (1.4-4.0)
**Cephalosporins**		
D1	1.6 (0.0-42.9)	Insufficient observations
D2	16.0 (8.2-28.9)	
D3-9	0.8 (0.2-3.5)	
**Positive post-therapy**		
Cephalosporin	10.4 (6.6-16.1)	No change (Brook *et al* had no post antibiotic results)
Macrolide	12.2 (7.6-19.0)	
Penicillin	14.3 (10.8-18.8)	

Supplementary Appendix G. Sensitivity analysis excluding studies that included participants with asymptomatic carriage (Edmond *et al*, Hoskins *et al* and Howie *et al*).

	All studies	Excluding studies with asymptomatic patients
**Overall**		
D1	6.9 (2.7-16.8)	6.0 (2.1-15.9)
D2	5.4 (2.1-13.3)	5.6 (1.9-15.3)
D3-9	2.6 (1.6-4.2)	2.3 (1.4-3.9)
**Penicillin**		
D1	6.5 (2.5-16.1)	5.6 (2.0-15.1)
D2	4.7 (1.7-12.4)	4.9 (1.5-14.5)
D3-9	2.6 (1.4-4.8)	2.5 (1.3-4.7)
**Cephalosporins**		
D1	1.6 (0.0-42.9)	No change (none of the asymptomatic studies had a cephalosporin arm)
D2	16.0 (8.2-28.9)	
D3-9	0.8 (0.2-3.5)	
**Positive post-therapy**		
Cephalosporin	10.4 (6.6-16.1)	10.4 (6.6-16.1)
Macrolide	12.2 (7.6-19.0)	14.0 (78.7-21.9)
Penicillin	14.3 (10.8-18.8)	14.3 (10.4-19.4)
Overall	13.3 (10.5-16.7)	13.4 (10.5-16.9)

References for supplementary material

1. Johnson DR KE, Sramek J, Bicova R, Havlicek J, Havlickova H, Motlova J, Kriz P; for World Health Organisation Geneva. Laboratory diagnosis of group A streptococcal infections. 1996.
2. Health NIo. Study quality assessment tools. . Bethesda: National Institutes of Health; 2020
3. Snellman LW, Stang HJ, Stang JM, Johnson DR, Kaplan EL. Duration of positive throat cultures for group A streptococci after initiation of antibiotic therapy. Pediatrics. 1993;91(6):1166-70.
4. Ryan DC, Dreher GH, Hurst JA. Estolate and stearate forms of erythromycin in the treatment of acute beta haemolytic streptococcal pharyngitis. Med J Aust. 1973;1(1):20-1.
5. Levine MK, Berman JD. A comparison of clindamycin and erythromycin in beta--hemolytic streptococcal infections. J Med Assoc Ga. 1972;61(3):108-11.
6. Watkins VS, Smietana M, Conforti PM, Sides GD, Huck W. Comparison of dirithromycin and penicillin for treatment of streptococcal pharyngitis. Antimicrob Agents Chemother. 1997;41(1):72-5.
7. Stein GE, Christensen S, Mummaw N. Comparative study of clarithromycin and penicillin V in the treatment of streptococcal pharyngitis. Eur J Clin Microbiol Infect Dis. 1991;10(11):949-53.
8. Ginsburg CM, McCracken GH, Jr., Steinberg JB, Crow SD, Dildy BF, Lancaster K, et al. Management of group A streptococcal pharyngitis: a randomized controlled study of twice-daily erythromycin ethylsuccinate versus erythromycin estolate. Pediatric Infectious Disease. 1982;1(6):384-7.
9. Lester RL, Howie VM, Ploussard JH. Treatment of streptococcal pharyngitis with different antibiotic regimens. Clinical Pediatrics. 1974;13(3):239-42.
10. Ginsburg CM, McCracken GH, Jr., Steinberg JB, Crow SD, Dildy BF, Cope F, et al. Treatment of Group A streptococcal pharyngitis in children. Results of a prospective, randomized study of four antimicrobial agents. Clin Pediatr (Phila). 1982;21(2):83-8.
11. Disney FA DM, Higgins JE, Nolen T, Poole JM, Randolph M, Rogan MP. . Comparison of once-daily cefadroxil and four-times-daily erythromycin in group A streptococcal tonsillopharyngitis. Advances in therapy. 1990;7(6):312-26.
12. De la Garza CA, Nolen TM, Rogan MP. Cefprozil vs. erythromycin in streptococcal tonsillopharyngitis. Infections in Medicine. 1992;9:8-20.
13. Sinanian R, Ruoff G, Panzer J, Atkinson W. Streptococcal pharyngitis: a comparison of the eradication of the organism by 5- and 10-day antibiotic therapy. Curr Ther Res Clin Exp. 1972;14(11):716-20.
14. Trickett PC, Dineen P, Mogabgab W. Clinical experience: respiratory tract. Trimethoprim-sulfamethoxazole versus penicillin G in the treatment of group A beta-hemolytic streptococcal pharyngitis and tonsillitis. J Infect Dis. 1973;128:Suppl:693-5 p.
15. Hoskins TW, Bernstein LS. Trimethoprim/sulphadiazine compared with penicillin V in the treatment of streptococcal throat infections. J Antimicrob Chemother. 1981;8(6):495-6.
16. Gerber MA, olph MF, DeMeo K, Feder HM, Jr., Kaplan EL. Failure of once-daily penicillin V therapy for streptococcal pharyngitis. American Journal of Diseases of Children. 1989;143(2):153-5.
17. Krober MS, Weir MR, Themelis NJ, van Hamont JE. Optimal dosing interval for penicillin treatment of streptococcal pharyngitis. Clinical Pediatrics. 1990;29(11):646-8.
18. Raz R EG, Colodner R, Reiss S, Schvartzman P, Tabenkin H, and Leshem Y. . Penicillin V twice daily vs. four times daily in the treatment of streptococcal pharyngitis. Infectious Diseases in Clinical Practice. 1995;4(1):50-4.
19. Feder HM, Jr., Gerber MA, olph MF, Stelmach PS, Kaplan EL. Once-daily therapy for streptococcal pharyngitis with amoxicillin. Pediatrics. 1999;103(1):47-51.
20. Shvartzman P, Tabenkin H, Rosentzwaig A, Dolginov F. Treatment of streptococcal pharyngitis with amoxycillin once a day. BMJ. 1993;306(6886):1170-2.
21. Lennon DR, Farrell E, Martin DR, Stewart JM. Once-daily amoxicillin versus twice-daily penicillin V in group A beta-haemolytic streptococcal pharyngitis. Archives of Disease in Childhood. 2008;93(6):474-8.
22. Colcher IS, Bass JW. Penicillin treatment of streptococcal pharyngitis. A comparison of schedules and the role of specific counseling. JAMA. 1972;222(6):657-9.
23. Ginsburg CM, McCracken GH, Jr., Steinberg JB, Crow SD, Dildy BF, Cope F, et al. Treatment of Group A streptococcal pharyngitis in children. Results of a prospective, randomized study of four antimicrobial agents. Clin Pediatr (Phila). 1982;21(2):83-8.

24. Pavesio D, Pecco P, Peisino MG. Short-term treatment of streptococcal tonsillitis with ceftriaxone. Chemotherapy. 1988;34 Suppl 1:34-8.
25. Randolph MF, Gerber MA, DeMeo KK, Wright L. Effect of antibiotic therapy on the clinical course of streptococcal pharyngitis. J Pediatr. 1985;106(6):870-5.
26. Dagnelie CF, van der Graaf Y, De Melker RA. Do patients with sore throat benefit from penicillin? A randomized double-blind placebo-controlled clinical trial with penicillin V in general practice. Br J Gen Pract. 1996;46(411):589-93.
27. Krober MS, Bass JW, Michels GN. Streptococcal pharyngitis. Placebo-controlled double-blind evaluation of clinical response to penicillin therapy. JAMA. 1985;253(9):1271-4.
